# Exploring Mechanistic Insights by Carotenoids in Neuropathic and Inflammatory Pain

**DOI:** 10.2174/011570159X371386250619064416

**Published:** 2025-07-02

**Authors:** Fatemeh Abbaszadeh, Masoumeh Jorjani, Roshanak Amirian, Sajad Fakhri, Haroon Khan

**Affiliations:** 1 Neurobiology Research Center, Institute of Neuroscience and Cognition, Shahid Beheshti University of Medical Sciences, Tehran, Iran;; 2 Department of Pharmacology, School of Medicine, Shahid Beheshti University of Medical Sciences, Tehran, Iran;; 3 Student Research Committee, Kermanshah University of Medical Sciences, Kermanshah, Iran;; 4 USERN Office, Kermanshah University of Medical Sciences, Kermanshah, Iran;; 5 Pharmaceutical Sciences Research Center, Health Institute, Kermanshah University of Medical Sciences, Kermanshah, Iran;; 6 Department of Pharmacy, Abdul Wali Khan University Mardan, Mardan, 23200, Pakistan;; 7 Department of Pharmacy, Korea University, Sejong, 20019, South Korea

**Keywords:** Carotenoids, inflammatory pain, neuropathic pain, signaling pathway, clinical profile, drug of future

## Abstract

Chronic pain, characterized by persistent discomfort and reduced quality of life, poses a significant challenge for individuals. Chronic pain is predominantly divided into central neuropathic pain, peripheral neuropathic pain, and inflammatory pain. Considering the multiple dysregulated pathways behind such pain conditions, researchers are exploring new multi-target agents that offer enhanced efficacy and reduced side effects of the present drugs. Carotenoids are natural pigments with antioxidant and anti-inflammatory properties found in various fruits, vegetables, and seafood. Through their mechanisms of action, carotenoids have shown promising efficacy in alleviating pain hypersensitivity, reducing inflammation and oxidative stress, and modulating pain-related signaling pathways. This comprehensive review delves into the potential of carotenoids and their derivatives as natural nutraceuticals for managing inflammation and relieving pain. In the current study, the mechanisms of action by which carotenoids exert their beneficial effects during preclinical and clinical studies are provided. This review could pave the road for the application of carotenoids for more pain-related clinical trials and further applications.

## 1 INTRODUCTION

Pathological pain also named chronic pain, is a type of long-lasting pain that persists for more than three months and is usually linked to a specific medical condition or disease. Unlike acute pain, which is a normal response to injury or disease and typically goes away once the underlying cause is treated or healed, pathological pain continues [1, 2]. Pathological pain is marked by its spontaneous onset without a clear or identifiable trigger, fluctuating intensity, increased sensitivity to painful stimuli known as hyperalgesia, and the experience of pain from non-painful stimuli, such as light touch, referred to as allodynia. This reflects an abnormal perception of pain [3]. There are three main categories of pathological pain: central neuropathic pain, peripheral neuropathic pain, and inflammatory pain [1, 2]. Neuropathic pain results from injury or dysfunction in the nervous system, such as nerve compression, diabetic neuropathy, or trauma-induced nerve damage [4]. Inflammatory pain occurs as a natural response to injury or infection, resulting in pain and swelling. Conditions like arthritis, where inflammation affects the joints, can cause persistent inflammatory pain [5, 6].

The pathway of pain signaling is a complex process that involves numerous interactions among peripheral and central nervous system (CNS) mediators. When tissue is injured, specialized sensory neurons called nociceptors detect harmful stimuli and transmit signals through the spinal cord to the brain, where pain is perceived [7]. The brain’s pain processing mechanisms involve complex interactions across various pathways, particularly the medial and lateral pathways. The lateral components of the ventral pathways, which encompass structures like the medial prefrontal cortex (mPFC), anterior cingulate cortex (ACC), insula, and certain areas of the thalamus, primarily regulate the emotional and affective aspects of pain. These regions modulate pain perception influenced by contextual factors, thereby affecting individuals’ emotional reactions to pain experiences. For instance, when pain is perceived as uncontrollable, the mPFC can heighten the emotional response, illustrating its role in merging sensory information with emotional context [8].

Furthermore, the central nucleus of the amygdala plays a role in integrating pain signals with emotional responses, linking pain perception to feelings of fear and anxiety. This highlights the significance of internal pathways in shaping the subjective experience of pain [9]. In contrast, the lateral pathways emphasize the sensory dimensions of pain processing. Key areas such as the primary somatosensory cortex (S1) and secondary somatosensory cortex (S2) are crucial for detecting the intensity and location of painful stimuli, forming part of what is referred to as the neuropathological pain signal. This pathway allows individuals to accurately localize pain and evaluate its intensity, which is essential for appropriate behavioral reactions [10].

Additionally, the dorsolateral prefrontal cortex (dlPFC) plays a vital role in the cognitive regulation of pain [8]. This signaling pathway involves various neurotransmitters, receptors, and modulatory systems, making it multifaceted and influenced by factors such as emotional state and previous experiences [7]. Accordingly, these pain-related disorders greatly affect an individual’s quality of life by causing physical discomfort and emotional distress while also hindering their capacity to engage in daily activities [11]. Given this complexity, effective pain management often necessitates multi-target techniques that address different aspects of the pain pathway simultaneously. These approaches may include pharmacological and nutritional interventions designed to target specific receptors and mechanisms to relieve pain and improve the overall quality of life for those experiencing chronic pain.

Among therapeutic candidates, carotenoids are a group of natural compounds known for their antioxidant and anti-inflammatory effects, which contribute to various biological functions, including pain relief [12, 13]. This review investigates the therapeutic potential of carotenoids and their derivatives as natural nutraceutical solutions for addressing the multifaceted challenges of chronic pain. It presents a comprehensive analysis of various pain conditions, including central/peripheral neuropathic and inflammatory pain, which affect millions of individuals worldwide. By summarizing the intricate mechanisms underlying the analgesic properties of carotenoids, this review aims to provide insight into how these versatile phytochemicals can influence the complex pathways involved in persistent pain. The review covers current preclinical and clinical studies that support the efficacy of carotenoids in pain management, offering a promising alternative to conventional treatments.

## 2 SEARCH STRATEGY

A comprehensive review was done to unveil the mechanistic role of carotenoids on neuropathic and inflammatory pain. Scholarly electronic databases were employed for the literature search, including Scopus, PubMed, and ScienceDirect, and English language articles were included through September 30, 2024.

The following keywords were employed for the search: (carotenoid*) and all carotenoid types in [title/abstract]) AND (neuropathic pain OR inflammatory pain) [title/abstract]. Two independent authors (F.A. and S.F.) did the search strategy, which was confirmed by the senior author (H.K.). However, based on the reference lists and the author’s expertise in included studies, additional related articles were added.

## 3 CLASSIFICATION OF CAROTENOIDS

Carotenes and xanthophylls are two major classes of carotenoids, distinguished by their chemical structures [14]. On the other hand, shorter molecules called apocarotenoids are produced from carotenoids like β-carotene, α-carotene, and zeaxanthin by enzymatic cleavage of double bonds in the polyisoprenoid chain [15].

### 3.1 Carotenes

Carotenes are hydrocarbons, meaning they consist solely of carbon and hydrogen atoms [14]. Some well-known examples of carotenes include β-carotene, α-carotene, and lycopene [16]. Since the human body cannot synthesize these compounds, they must be acquired through dietary sources or supplements [17]. Carotenoids are predominantly found in algae, plants, and photosynthetic bacteria, giving rise to the striking yellow, red, and orange hues present in foods such as carrots, tomatoes, sweet potatoes, kale, spinach, and citrus fruits [18]. β-carotene is a linear tetraterpene with the chemical formula C_40_H_56_. It consists of two symmetrical halves, each containing a β-ring at the ends. The structure features a long chain of alternating double bonds, contributing to its color and antioxidant properties. The extensive system of conjugated double bonds allows β-carotene to absorb light effectively, giving it its characteristic orange color [19]. α-Carotene is similar to β-carotene in that it also has the formula C_40_H_56_ but differs in the presence of an ε-ring at one end instead of a β-ring. This structural difference affects its conversion efficiency to vitamin A and its biological activity. Like β-carotene, α-carotene contains a long chain of conjugated double bonds, which also contributes to its pigmentation and antioxidant properties [20]. Lycopene is a linear carotenoid with the same molecular formula C_40_H_56_ but lacks the rings present in β-carotene and α-carotene. Instead, it has a series of conjugated double bonds without any cyclic structures. This unique configuration gives lycopene its deep red color and makes it a powerful antioxidant [19, 21].

### 3.2 Xanthophylls

Xanthophylls are carotenoids that contain oxygen atoms in addition to carbon and hydrogen. This oxygenation gives them a slightly different chemical structure compared to carotenes. Lutein, zeaxanthin, and astaxanthin are examples of xanthophylls, and they are often found in green leafy vegetables and certain seafood [22]. Lutein is a xanthophyll with the chemical formula C_40_H_56_O_2_. It has a molecular weight of approximately 568.87 g/mol and features a long polyene chain with conjugated double bonds, which gives it distinctive light-absorbing properties. The principal stereoisomer of lutein is (3R,3′R,6′R)-beta,epsilon-carotene-3,3′-diol. Lutein is primarily synthesized by plants and is abundant in green leafy vegetables such as spinach and kale, where it plays a role in photosynthesis by modulating light energy and protecting against photodamage [23, 24]. Zeaxanthin shares a similar structure with lutein but differs in the arrangement of double bonds; its chemical formula is also C_40_H_56_O_2_. It has been shown to reduce the risk of age-related macular degeneration (AMD) and other eye diseases [25, 26]. The presence of both lutein and zeaxanthin in the diet is often recommended for optimal eye health [23]. Astaxanthin is another carotenoid with a chemical formula of C_40_H_52_O_4_. Distinguishing it from lutein and zeaxanthin due to its additional oxygen atoms. It has a unique structure that includes two ionone rings connected by a polyene chain. Astaxanthin is known for its deep red color and is synthesized by various microalgae, yeast, and seafood. It protects cells from oxidative damage and inflammation [25].

### 3.3 Apocarotenoids

Apocarotenoids are a diverse group of compounds derived from, like β-carotene, α-carotene, and zeaxanthin, characterized by the oxidative cleavage of their carbon-carbon double bonds. This process can occur enzymatically through carotenoid cleavage dioxygenases (CCDs) or non-enzymatically *via* reactive oxygen species (ROS) [26]. The resulting apocarotenoids, such as crocin, crocetin, bixin, picrocrocin, and safranal, exhibit various biological functions and properties [15]. The structural characteristics of apocarotenoids contribute to their functional diversity. They typically possess a shortened carbon skeleton compared to their parent carotenoids, leading to variations in solubility and reactivity. For example, crocin (C_22_H_20_O_11_) is known for its vibrant yellow color and potential health benefits, including antioxidant and anti-inflammatory effects. Crocetin (C_22_H_24_O_8_), a dicarboxylic acid derivative of crocin, contributing to the color and flavor of saffron. Bixin (C_27_H_34_O_4_), another apocarotenoid, is primarily found in annatto seeds and is used as a natural food colorant due to its lipophilic nature. Safranal (C_13_H_10_O), derived from saffron, not only imparts flavor and aroma but also exhibits neuroprotective properties. Picrocrocin (C_16_H_26_O_7_), the precursor to safranal found in saffron, is responsible for its bitter taste and has been studied for its potential therapeutic effects. The structural modifications during the cleavage process often result in compounds that can interact with biological systems in unique ways [27].

Regular dietary intake from these natural sources is linked to numerous health benefits without significant risk, whereas excessive supplementation may lead to adverse effects, especially among vulnerable populations like smokers [28].

## 4 CAROTENOIDS AND NEUROPATHIC PAIN

### 4.1 Carotenoids and Central Neuropathic Pain

Central neuropathic pain, also known as central pain syndrome, refers to a type of chronic pain that originates from damage or dysfunction in the CNS, particularly the brain or spinal cord. It can occur as a result of various conditions or injuries that affect the CNS, such as spinal cord injury (SCI), multiple sclerosis (MS), stroke, or brain trauma [29]. The processes such as changes in neuronal excitability, neurotransmitter release, and ion channel/receptor signatures (*e.g*., N-methyl-D-aspartate (NMDA) receptors and voltage-gated sodium channels), contribute to the onset and continuation of neuropathic pain [30, 31].

When central sensitization occurs, the threshold for activation of neurons decreases, meaning that they become more easily activated. This can lower the pain threshold, causing even mild or non-painful stimuli to be experienced as painful. Additionally, the strength of synaptic connections among neurons is enhanced, which means that signals related to pain can be amplified and transmitted more efficiently [32, 33]. Another reason for increased pain sensitivity is reactive gliosis, which involves the activation and proliferation of glial cells. Glial cells, especially astrocytes and microglia, secrete chemicals such as cytokines, chemokines, and growth factors that cause inflammation and pain. These products can sensitize neurons, leading to further transmission and development of neuropathic pain [34]. Moreover, oxidative stress can contribute to central sensitization by enhancing neuroinflammation, elevating neuronal excitability and neurotransmitter release, and disrupting natural pain regulation [35]. Oxidative stress promotes the release of glutamate in the spinal cord and brain, activating NMDA receptors that play a role in synaptic plasticity and neuronal connection reinforcement.

Additionally, substance P, a neuropeptide involved in pain transmission, is potentiated by the sensitization of the transient receptor potential (TRP) vanilloid 1 (TRPV1) channel, which can be influenced by oxidative stress [35, 36]. The activation of these types of calcium channels (TRPVs) by oxidative stress and the subsequent increase in calcium influx can have several effects on neuronal excitability, amplification of pain signals, and the development and maintenance of central sensitization. The increased calcium influx, firstly, can activate intracellular signaling pathways that enhance the sensitivity of ion channels, including sodium channels, leading to increased excitability [37]. Secondly, calcium can activate various enzymes, such as protein kinases and phosphatases, which can further modulate ion channel function and neuronal excitability. Thirdly, calcium can trigger the release of neurotransmitters from presynaptic terminals, leading to increased synaptic transmission and neuronal activity [37, 38].

Overall, central neuropathic pain is a multifaceted condition arising from dysfunction within the CNS, characterized by alterations in neuronal excitability, neurotransmitter release, and the signatures of ion channels and receptors. Key processes such as central sensitization, reactive gliosis, and oxidative stress significantly contribute to the amplification of pain signals, leading to the development and persistence of neuropathic pain. Emerging evidence suggests that carotenoids effectively regulate these mechanisms, offering a promising approach to managing central neuropathic pain (Table **1**).

#### 4.1.1 Carotenoids and Spinal Cord Injury Pain

Up to 80% of individuals with SCI experience significant neuropathic pain [39] due to complex pathophysiological mechanisms involving neurochemical, excitotoxic, and inflammatory changes in the CNS. These alterations can lead to heightened pain processing, resulting in conditions like allodynia and hyperalgesia [30, 40].

Over the years, studies have shown that the xanthophyll compound astaxanthin, which has potent antioxidant properties, has promising effects in improving sensorimotor function in spinal cord injury (SCI), especially in reducing neuropathic pain. This pain reduction is linked to the downregulation of key inflammatory signaling mediators such as NMDA receptor subunit 2B (NR2B) and p-p38 mitogen-activated protein kinase (MAPK) [41], as well as the decrease in levels of inflammatory cytokines like cyclooxygenase 2 (COX-2), tumor necrosis factor-alpha (TNF-α), interleukin-1 beta (IL-1β), and IL-6 [41, 42]. Astaxanthin also helps in mitigating oxidative stress, reducing edema, and suppressing astrocyte activation through the inhibition of the high mobility group box 1 (HMGB1)/toll-like receptor 4 (TLR4)/NF-κB signaling pathway [43]. Furthermore, it protects spinal cord tissues from apoptosis by modulating Bax, Bcl, and caspase levels [44], maintains the integrity of the blood-spinal cord barrier by regulating matrix-metalloproteinase 9 (MMP9) [43], and influences autophagy, neuronal oxidative stress [45], extracellular signal-regulated kinases (ERKs), and protein kinase B (AKT) signaling pathways [46]. Additionally, astaxanthin decreases the expression of macrophage migration inhibitory factor (MIF) [47] and promotes mitochondrial biogenesis [48]. Li *et al.*'s findings also confirmed our results regarding the protective effects of astaxanthin on spinal cord tissue [49]. One study conducted on rats with SCI reported that astaxanthin treatment significantly increased the expression of neurotrophin-3 (NT-3) in the injured spinal cord [50]. NT-3 has been found to suppress thermal hyperalgesia associated with neuropathic pain and attenuate TRPV1, a key receptor involved in pain perception [51]. These results indicate that astaxanthin may influence important pathways related to the onset and persistence of neuropathic pain following SCI.

From the category of carotene, it has been reported that β-carotene helped reduce oxidative stress caused by SCI by regulating ROS, malondialdehyde (MDA), nitric oxide (NO), and superoxide dismutase (SOD) levels, and restored reduced protein expressions of Nuclear factor erythroid 2-related factor 2 (Nrf2) and heme oxygenase-1 (HO-1) due to SCI. Additionally, β-carotene decreased the production of pro-inflammatory cytokines like TNF-α, IL-1β, IL-18, and COX-2, inhibited the NF-κB pathway, and reduced astrocyte activation in the spinal cord [52]. Lycopene, another member of this family, also showed similar effects. Lycopene reduced the SCI-induced elevation in the protein levels of COX-2, NF-κB, and activated protein1 (AP1) and also prevented the decrease in HO-1 [53]. When it was administered to rats at a dose of 4 mg/kg/day immediately after SCI, it remarkably reduced edema in the spinal cord tissue and enhanced the integrity of the blood–spinal cord barrier (BSCB) compared to untreated injured rodents. Furthermore, treatment with lycopene also upregulated proteins in the BSCB (zonula occluden-1 and claudin-5) and downregulated the levels of pro-inflammatory cytokines TNF-α and Nuclear factor kappa B (NF-κB) [54]. Another study on rats with T10 contusion SCI showed that lycopene treatment effectively reduced oxidative damage, improved mitochondrial function, and decreased cell apoptosis [55]. Combining lutein’s antioxidant activity and anti-inflammatory effects can help support spinal cord neurons and mitigate the damage caused by ischemia-reperfusion injury [56]. Studies have provided evidence that apocarotenoids such as crocin can decrease calcitonin gene expression, contributing to its beneficial effects on chronic pain induced by SCI in rat models [57].

#### 4.1.2 Carotenoids and Stroke-induced Neuropathic Pain

Neuropathic pain after stroke, known as central post-stroke pain (CPSP), affects 1-12% of survivors [58], typically emerging 3 to 6 months post-stroke [59]. It is often linked to conditions like myofascial pain syndrome and is characterized by muscle tension and trigger points. The exact cause is unclear, but factors such as spinothalamic tract dysfunction and hyperactivity of the thalamus are believed to contribute. Neurotransmitters like tachykinins, calcitonin gene-related peptides, and glutamate are involved in the pain mechanisms through excitatory effects and increased sensitization [58, 60].

The neuroprotective effects of astaxanthin in both *in vitro* and *in vivo* stroke models are significant, making it a promising therapeutic agent [61]. The mechanisms of astaxanthin in protecting against free radical damage and neurodegeneration induced by ischemia/reperfusion in rats include the suppression of ROS, activation of the Nrf2–ARE defense pathway, prevention of apoptosis, and promotion of neural regeneration [62]. According to research, taking 45 mg/kg of astaxanthin has a significant impact on normalizing the total oxidant status (TOS) and the levels of caspase-3, Bax, and Bcl2, the activity of antioxidant enzymes glutathione peroxidase (GPx), catalase (CAT), and NF-κB [63]. Moreover, the findings suggested that astaxanthin has the potential to improve acute cerebral infarction by reducing oxidative stress and increasing the expression of brain-derived neurotrophic factor (BDNF) and nerve growth factor (NGF) mRNA [64]. Yang *et al.* also confirmed the protective effects of astaxanthin against acute cerebral infarction. Their findings suggest that these effects may be attributed to the activation of Nrf2/HO-1 signaling, which suppresses oxidative stress, inflammation, and apoptosis [65].

Additionally, astaxanthin has the potential to decrease ischemia-induced brain tissue damage by inhibiting the release of glutamate [66] and downregulation of p75 neurotrophin receptor expression [67]. The effects of astaxanthin on axonal regeneration in mice with focal cerebral infarction involves the activation of the cAMP/PKA/CREB signaling pathway by increasing the cAMP concentration in brain tissues [68]. The administration of 0.2 mg/kg of lutein, another member of the xanthophyll family, to mice with cerebral ischemia/reperfusion injury has been found to have anti-apoptotic, anti-oxidative, and anti-inflammatory impacts through modulation of phosphoinositide 3-kinas (PI3K)/ AKT, MAPK/ERK, and NF-κB signaling pathways [69].

The neuroprotective impact of carotenoids like β-carotene following brain injury is associated with the upregulation of genes involved in cholesterol regulation, such as Apo E, and the promotion of neuronal repair [70]. Wei *et al.* reported that the protective effects of lycopene against ischemia-reperfusion injury may be related to its ability to reduce the accumulation of ROS and lactic acid, nitric oxide synthetase, and increase the expression of hypoxia-inducible factor 1α (HIF-1α) and Bcl-2 mRNA genes [71]. On the other hand, the use of nanoliposomes containing lycopene has various beneficial molecular effects in reducing ischemic brain damage. It effectively decreases the level of oxidase while simultaneously increasing the level of Bcl-2, decreasing the level of caspase-3, and inhibiting apoptosis through the suppression of cell death pathways such as MAPK- c-Jun NH2-terminal kinase (JNK) [72].

The antioxidant properties of crocin and safranal of the apocarotenoids family have also been reported to be effective in mitigating pain and pathophysiology changes following stroke. In the early stage of intracerebral hemorrhage (ICH), the dosage of 40 mg/kg of crocin resulted in the alleviation of iron deposition, myelin loss, ROS production, HO-1 expression, and neuron degeneration [73]. In a mouse model of ICH, the administration of crocin resulted in a reduction in brain edema and neurological deficits. Furthermore, crocin treatment led to an increase in the activity of SOD and GPx, while decreasing MDA levels. Crocin effectively inhibited neuronal ferroptosis by elevating the concentration of Fe^2+^ and upregulating the expression of ferroptosis-related genes such as ferritin heavy chain (FTH1), GP_X_4, and solute carrier family 7 member 11 (SLC7A11) [74].

**Fig. (1):**
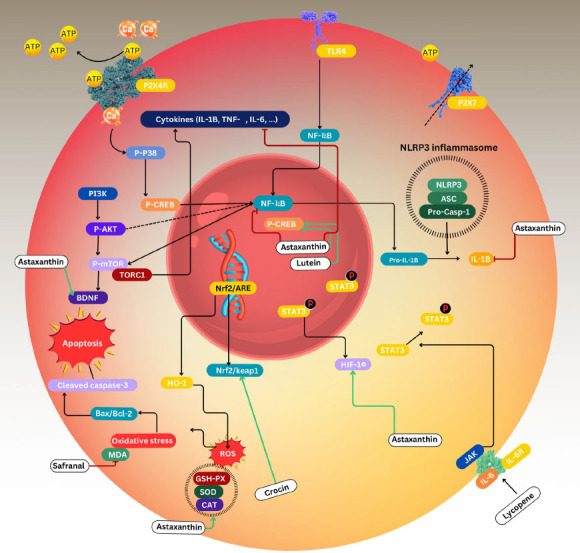
Molecular pathways mediating carotenoid effects on neuropathic pain in models of stroke. **Abbreviations**: Apoptosis-associated speck-like protein containing a CARD (ASC), Antioxidant response element (ARE), Brain-derived neurotrophic factor (BDNF), B-cell lymphoma protein 2 (Bcl-2),-associated X (Bax), Catalase (CAT), cAMP-response element binding protein (CREB), Glutathione (GSH), Hypoxia-inducible factor 1α (HIF-1α), Heme oxygenase-1 (HO-1), Interleukin (IL), Janus kinase (JAK), Kelch-like ECH-associated protein 1 (Keap1), Malondialdehyde (MDA), Mammalian target of rapamycin (mTOR), Nuclear factor kappa B (NF-κB), NOD-like receptor protein 3 (NLRP3), Nuclear factor erythroid 2-related factor 2 (Nrf2), Protein kinase B (AKT), Phosphatidylinositol 3 kinase (PI3K), Purinergic receptor P2X7 (P2X7R), Reactive oxygen species (ROS), Superoxide dismutase (SOD), Signal transducer and activator of transcription 3 (STAT3), Toll-like receptor (TLR), Tumor necrosis factor-alpha (TNF-α), Transducer of regulated CREB-activity (TORC).

On the other hand, crocin's ability to inhibit autophagy through the mammalian target of rapamycin (mTOR)/AMPK/Unc-51-like autophagy activating kinase 1 (ULK1) pathway contributes to its neuroprotective effects in cerebral ischemia by preventing apoptotic cell death and reducing ischemic damage [75]. In an animal study, crocin pretreatment significantly reduced the volume of brain infarction and brain water content while also enhancing neurological function. The protective effects of crocin were attributed to the preservation of tight junction proteins, reduction in nicotinamide adenine dinucleotide phosphate (NADPH) oxidase levels, and inhibition of matrix metalloproteinases [76]. The neuroprotective effects of crocin have been confirmed by various other studies [77-80]. Safranal has been found to attenuate cerebral ischemia-induced oxidative damage in the rat hippocampus by reducing lipid peroxidation, increasing antioxidant capacity, and elevating total sulfhydryl contents [81]. A study found that focal cerebral ischemia resulted in significant negative effects, including increased neurological impairment, larger tissue death in the brain, loss of neuronal cells in specific brain regions, and increased oxidative stress. However, when safranal was administered, these effects were reversed (Fig. **1**) [82].

#### 4.1.3 Carotenoids and Multiple Sclerosis-induced Neuropathic Pain

Neuropathic pain is a prevalent and distressing symptom in MS, significantly impacting patients’ quality of life and associated with disability and depression. It arises from neuroinflammation and axonal damage due to immune cell infiltration, particularly T cells, which attack myelin and disrupt nerve signal transmission [83]. This demyelination leads to various pain types, including trigeminal neuralgia, Lhermitte’s phenomenon, and dysaesthetic extremity pain, characterized by burning or tingling sensations. Understanding these pain conditions is essential for developing effective treatment strategies to enhance pain relief for MS patients [84, 85].

New research indicates that individuals with MS may exhibit changes in their carotenoid levels. Specifically, studies have found that MS patients have a deficiency of certain carotenoids, including lutein and zeaxanthin, compared to those who are healthy. These fluctuations in carotenoid levels may be connected to the inflammatory processes that occur in MS. The convenience of measuring and analyzing carotenoids makes them a promising option for diagnosing MS [86]. Conversely, research suggests that carotenoid treatment can decrease the pathology of MS. Studies have shown that astaxanthin can induce the differentiation of human Adipose-Derived Stem Cells (hADSCs) into oligodendrocyte precursor cells, which are crucial for myelination in the CNS [87]. An animal study evaluated the protective effects of astaxanthin against demyelination and the death of oligodendrocytes in an MS rat model. The results showed that administering 3 mg/kg of astaxanthin daily for 4 weeks reduced oligodendrocyte damage and disruption of myelin sheaths, leading to enhanced muscle strength [88]. Another study examined the preventive and therapeutic effects of astaxanthin in the experimental autoimmune encephalomyelitis (EAE) mouse model of MS and found that astaxanthin was effective in reducing the severity of EAE symptoms [89].

**Fig. (2):**
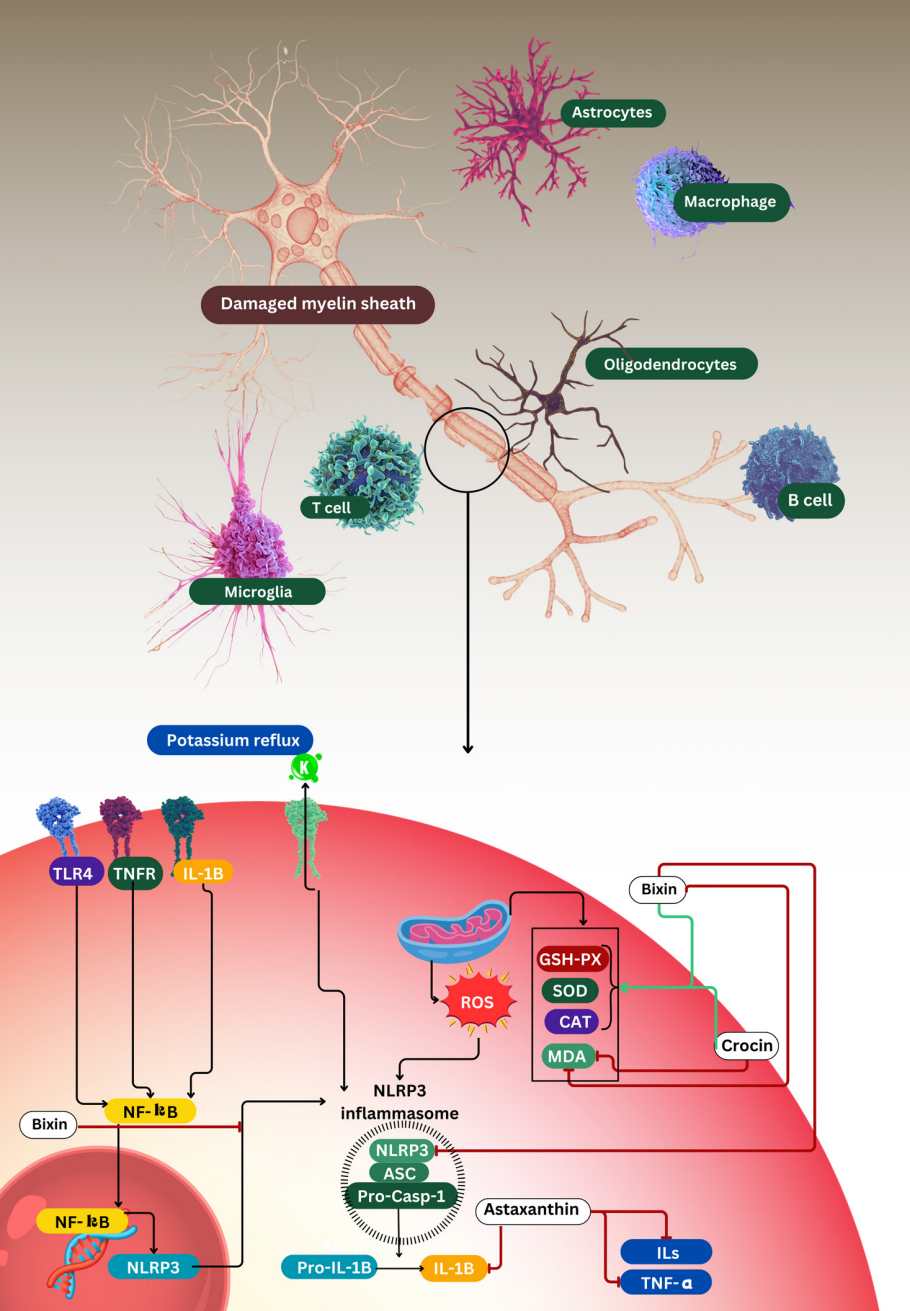
Molecular pathways mediating carotenoid effects on neuropathic pain in models of MS. **Abbreviations**: Apoptosis-associated speck-like protein containing a CARD (ASC), Catalase (CAT), Glutathione (GSH), Interleukin (IL), Malondialdehyde (MDA), Multiple sclerosis (MS), Nuclear factor kappa B (NF-κB), NOD-like receptor protein 3 (NLRP3), Reactive oxygen species (ROS), Superoxide dismutase (SOD), Toll-like receptor (TLR), Tumor necrosis factor-alpha (TNF-α), TNF receptor (TNFR).

Crocin treatment displayed remarkable efficacy in inhibiting endoplasmic reticulum stress and suppressing inflammatory gene expression in the spinal cords. This positive impact was further supported by the preservation of myelination and axonal density, as well as a reduction in T-cell infiltration and macrophage activation. Notably, crocin treatment improved the neurobehavioral deficits associated with EAE [90]. Crocin pretreatment in a mouse model of MS resulted in a decrease in MDA production and an increase in GPx, SOD, and total antioxidant status (TAS) levels in both serum and brain tissue [91]. In a study on EAE mice, the oral administration of bixin as an apocarotenoid significantly impacted symptoms and pathology positively. It effectively decreased the release of inflammatory cytokines such as TNF-α, IFN-γ, IL-6, IL-17, and IL-8 while increasing the levels of the anti-inflammatory cytokine IL-10. Bixin also reduced the proportion of Th1 and Th17 cells in both the spleen and CNS, and it hindered the accumulation of microglia while suppressing the activity of thioredoxin-interacting protein (TXNIP)/NOD-, LRR- and pyrin domain-containing protein 3 (NLRP3) inflammasome through the elimination of excessive ROS. Additionally, bixin activated Nrf2 and its downstream genes, mitigating inflammation and oxidative stress in EAE mice [92]. Another study reviewed the therapeutic potential of bixin in reducing inflammation, emphasizing its effectiveness in mitigating demyelination and axonal degeneration in mice with EAE (Fig. **2**) [93].

#### 4.1.4 Carotenoids and Traumatic Brain Injury-induced Neuropathic Pain

Neuropathic pain frequently occurs after traumatic brain injury (TBI), affecting over 50% of individuals with chronic pain [94]. Following TBI, complex pathophysiological processes are initiated, including neuroinflammation, neurodegeneration, and axonal damage, which contribute to chronic pain development. Neuroinflammation, characterized by elevated cytokines like TNF-α and IL-1β in pain-regulating brain areas, plays a significant role in this process [95]. Additionally, TBI is linked to neurodegenerative disorders such as Parkinson’s and Alzheimer’s diseases, primarily due to diffuse axonal injury (DAI) caused by traumatic forces. Chemokine signaling and structural and biochemical changes in the brain also affect synaptic function, which may exacerbate pain experiences [95-97]. Understanding these mechanisms is crucial for addressing chronic pain in TBI patients and preventing long-term neurodegenerative disorders.

Multiple studies suggest that carotenoids are effective in addressing these factors and alleviating pain. Astaxanthin can reduce oxidative stress and neuronal apoptosis, providing neuroprotective effects against TBI through the Sirtuin 1 (SIRT1)/p38 signaling pathway [98]. Astaxanthin also restored the levels of growth-associated protein-43 (GAP-43), BDNF, synaptophysin, and synapsin I in the cerebral cortex, leading to enhanced sensorimotor function after moderate TBI in animal models [99, 100]. Gao and his colleagues have also attributed these effects to the heightened activation of proteins, including Nrf2, HO-1, NAD(P)H quinone dehydrogenase 1 (NQO1), and SOD1. Additionally, they have revealed the anti-apoptotic properties of astaxanthin as a contributing factor [101]. On the other hand, the available evidence indicates that astaxanthin alleviates cerebral edema following TBI by modulating the expression of two key proteins involved in edema formation – Na-K-Cl cotransporter (NKCC) and aquaporin-4 (AQP4) [102]. Research has shown that when lutein/zeaxanthin isomers are given immediately after TBI, they reduce the size of the damaged area, improve the integrity of the blood-brain barrier, and lower the levels of inflammatory molecules like IL-1β, IL-6, and NF-κB. Moreover, this treatment enhances the levels of GAP-43, BDNF, and Nrf2, promoting the growth of neural cells [103]. In another study, treatment of TBI rats with lutein was associated with decreased levels of inflammatory biomarkers such as IL-1β, IL-6, monocyte chemoattractant protein 1 (MCP-1), and intercellular adhesion molecule-1 (ICAM-1) as well as modulation of oxidative stress factors like ROS, SOD, and glutathione (GSH) [104].

**Fig. (3):**
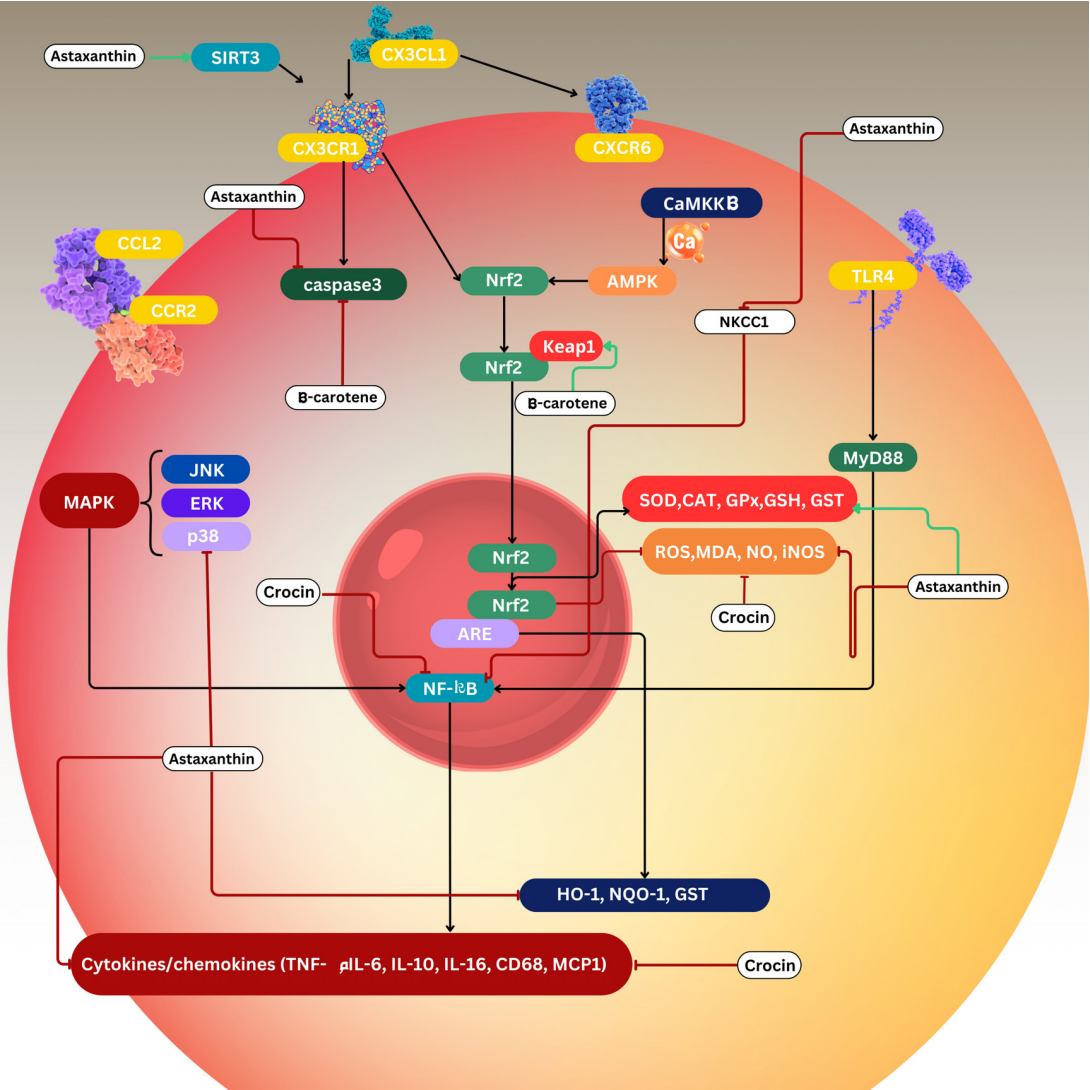
Molecular pathways mediating carotenoid effects on neuropathic pain in models of TBI. **Abbreviations**: Antioxidant response element (ARE), Brain-derived neurotrophic factor (BDNF), Catalase (CAT), c-Jun N-terminal kinase (JNK), chemokine (C-C motif) ligand 2 (CCL2), CXC chemokine receptor (CXCR), Extracellular-signal regulated kinase (ERK), Glutathione (GSH), Glutathione-S-Transferase (GST), Heme oxygenase-1 (HO-1), Interleukin (IL), Janus kinase (JAK), Kelch-like ECH-associated protein 1 (Keap1), Malondialdehyde (MDA), Mitogen-activated protein kinases (MAPKs), Nuclear factor kappa B (NF-κB), NOD-like receptor protein 3 (NLRP3), NADPH Quinone Oxidoreductase (NQO1), Nuclear factor erythroid 2-related factor 2 (Nrf2), Reactive oxygen species (ROS), Superoxide dismutase (SOD), Sirtuin 1 (SIRT1), Sodium Potassium Chloride Cotransporter 1 (NKCC1), Toll-like receptor (TLR), Tumor necrosis factor-alpha (TNF-α), Traumatic brain injury (TBI).

In terms of the effect on the oxidative stress pathway, Chen *et al.* reported that β-carotene exerts its neuroprotective effects after TBI through the activation of the Nrf2-ARE pathway, leading to increased antioxidant defense, reduced oxidative stress, and suppression of neuroinflammation [105]. Treatment with apocarotenoids like crocetin significantly improved neurological dysfunctions and reduced brain edema in mice after experiencing TBI. Moreover, crocetin exhibited remarkable effects by reducing neuronal apoptosis and neuroinflammation while also enhancing autophagy in the mouse brain [106]. By reducing oxidative stress, inflammatory responses, and behavioral deficits, saffron extract and crocin demonstrate a neuroprotective impact in a mouse model of TBI (Fig. **3**) [107].

### 4.2 Carotenoids and Peripheral Neuropathic Pain

Peripheral neuropathic pain is a form of chronic pain that occurs due to damage or disease impacting the peripheral nervous system. This can happen in conditions such as diabetic neuropathy, nerve compression, or traumatic injuries. The peripheral nervous system plays a crucial role in conveying sensory information from the body to the CNS, and when it is damaged, it can result in abnormal pain signals being sent to the brain [109]. Also, peripheral nervous system pain can be caused by several factors, including hypoxia, trauma, overload, compression, inflammation, and chemical factors that can damage the axon and promote pathological changes [110]. The peripheral mechanisms in neuropathic pain involve a complex interplay of factors within the peripheral nervous system that contribute to the development and maintenance of neuropathic pain. Following a peripheral nerve injury, a cascade of events occurs in primary afferents and dorsal root ganglion (DRG), leading to peripheral sensitization. This process involves the hyperexcitability of sensory neurons resulting from reduced thresholds and an enhanced response following nerve or tissue injury, inflammation, and the release of pro-nociceptive mediators. These mediators include histamine, serotonin, bradykinin, substance P, prostaglandins, and cytokines, all of which contribute to the heightened perception of pain [110, 111]. On the other hand, The peripheral nerve endings of unmyelinated C fibers and thinly myelinated Aδ fibers can be affected by inflammatory mediators, medications, and toxins, resulting in disrupted signaling and pain transmission [112].

Recent research has highlighted the role of non-neuronal cells in the peripheral nervous system, such as Schwann cells and macrophages, in releasing substances that contribute to the development of neuropathic pain [113, 114]. Upon peripheral nerve injury, Schwann cells and macrophages release arachidonic acid and its derivatives, primarily prostaglandins (PGs). These PGs play a role in regulating the function of peripheral sensory nerves through paracrine and autocrine mechanisms, contributing to peripheral sensitization and hyperalgesia [115]. In particular, prostaglandin E2 (PGE2) and its prostaglandin E receptors (EPs) play a significant role in neuropathic pain. Schwann cells express and upregulate various receptors and ion channels, such as purinergic receptors, toll-like receptors (TLRs), transient receptor potential ankyrin 1 (TRPA1), gamma-aminobutyric acid receptor B (GABABR), and acetylcholine (ACh) receptors, all of which are crucial for nociception. Exogenous molecules released from damaged tissues, immune cells, and neurons interact with specific receptors on the membranes of Schwann cells and promote cytokines, chemokines, and other pro-inflammatory substances, further contributing to peripheral sensitization and neuropathic pain [110, 116]. Schwann cell TRPA1 is involved in neuroinflammation that perpetuates macrophage-dependent neuropathic pain in mice [117]. The interplay between Schwann cells and macrophages is vital for understanding neuropathic pain, particularly in the context of peripheral nerve injuries and inherited demyelinating diseases, where macrophage activity and Schwann cell responses are key to axon regeneration [116].

Additionally, changes in ion channels are observed in the development of neuropathic pain. This includes the synthesis of rapidly repriming sodium channels, down-regulation of Tetrodotoxin (TTX)-resistant sodium channels, and loss of high-voltage activated N-type calcium channels [118]. In addition, there is an increase in the expression of the calcium-activated chloride channel TMEM16A in the neurons of the DRG [119]. This channel amplifies action potentials and enhances pain signals triggered by the activation of sensory neuronal channels and receptors. Consequently, hyperexcitability and persistent hypersensitivity to pain are observed [120, 121].

Furthermore, the upregulation of CCR2 and TNF receptor-associated factor 6 (TRAF6) expression in the dorsal horn of the spinal cord is vital for the onset and persistence of neuropathic pain. These molecules operate through CCL2/CCR2-dependent mechanisms and NMDAR-mediated central sensitization. This upregulation contributes to the increased sensitivity to pain experienced in neuropathic pain conditions [122].

The chronic constriction injury (CCI) model (partially ligating the sciatic nerve), the partial sciatic nerve ligation (PSNL) model (tightly ligating a portion of the sciatic nerve), and the spinal nerve ligation (SNL) model (tightly ligating the L5 spinal nerve) are three commonly used surgical models of peripheral neuropathic pain in animal models like mice and rats [123].

The administration of astaxanthin demonstrated a reduction in mechanical and thermal hypersensitivity in rats with CCI-induced neuropathy [124]. Zhang *et al.* attributed the effectiveness of astaxanthin in reducing neuropathic pain after CCI to regulating HO-1 expression in the spinal cord and suppressing oxidative stress and inflammatory responses like TNF-α and IL-1β [125]. On the other hand, astaxanthin, through Nrf2 activation and inhibition of all three MAPKs (p38, ERK, JNK), provided pain relief and showed promise for neuropathic pain treatment in the CCI model [126]. The administration of trans-astaxanthin at a dosage of 80 mg/kg effectively countered the heightened expression of IL-1β, IL-6, and TNF-α in the hippocampus and spinal cord of rats with CCI. This led to an improvement in thermal hyperalgesia and the associated depression-like behaviors in these rats [127]. Studies proposed that administering saffron and crocin following CCI could potentially lead to elevated thresholds for mechanical and thermal pain [128-130]. The cannabinoid receptor is one of the possible mechanisms by which crocin exerts its analgesic effects [131]. Crocin injection effectively enhanced the analgesic effect of morphine in CCI rats by decreasing mechanical allodynia and lowering serum BDNF levels [132]. Intracerebroventricular (ICV) administration of crocin, in conjunction with its interaction with α2-adrenoceptors, can ameliorate memory deficits and improve hippocampal synaptic plasticity in animal models of CCI [133]. The behavior test results suggested astaxanthin may effectively relieve pain in SNL mice. Astaxanthin appeared to lower neuronal and non-neuronal activation levels, downregulate inflammatory pathway mediators like p-ERK1/2, p-p38 MAPK, and NF-κB, and reduce expression of pro-inflammatory cytokines such as IL-1β, IL-17, IL-6, and TNF-α [134]. Downregulation of connexin 43 (Cx43) is linked to the development of neuropathic pain after nerve injury, so restoring normal Cx43 levels can help reduce pain. The evidence suggests that lycopene can upregulate TNF-induced downregulation of Cx43 protein expression in spinal astrocytes through a transcription-independent mechanism. This modulation of Cx43 expression by lycopene contributes to the restoration of normal spinal Cx43 levels, which is significant in reducing the SNL model-induced pain [135]. The hyperalgesia induced by PSNL in rats was significantly reduced after a 14-day regular lycopene administration. This reduction was attributed to the increased activity of SOD and MDA, as well as the decreased level of GPx [136] (Table **2**).

The causes of peripheral neuropathic pain are diverse and can stem from various sources, such as hereditary, metabolic, infectious, and chemotherapy-induced peripheral neuropathy [109, 137].

#### 4.2.1 Carotenoids and Metabolic Neuropathies

Metabolic neuropathies are nerve disorders caused by diseases that disrupt the body’s chemical processes. They may result from energy utilization issues due to nutritional deficiencies or the accumulation of toxins [138]. Diabetes is the main cause, and other causes include alcohol consumption disorder, liver or kidney disease, and lack of B and E vitamins [139].

Diabetic neuropathy manifests as nerve damage due to high blood sugar, leading to various symptoms depending on the type of neuropathy, such as focal or polyneuropathy [140]. The condition arises from systemic imbalances like oxidative stress and inflammation, which damage nerves and impair their function. Chronic inflammation exacerbates diabetic peripheral neuropathy by disrupting insulin signaling and promoting cytokine production, which hinders tissue repair. Additionally, obesity increases the risk of neuropathy through elevated levels of inflammatory markers associated with insulin resistance [141-143].

The anti-inflammatory and antioxidant attributes of carotenoids have been noted for their role in alleviating these symptoms. Astaxanthin effectively suppresses hyperglycemia by increasing insulin sensitivity, AMPK signaling, adiponectin concentration, and insulin concentration [144]. In a study conducted on streptozotocin (STZ)-induced diabetic rats, lycopene was found to alleviate thermal hyperalgesia (increased sensitivity to heat) and cold allodynia (pain caused by cold stimuli) [145]. These properties may arise from its potential to inhibit the release of NO and TNF-α [146]. According to an animal study, lycopene appeared to have a protective effect against diabetic optic neuropathy. This effect was linked to the regulation of oxidative stress markers such as MDA, TOS, oxidative stress index (OSI), TGSH, and TAS, as well as the reduction of inflammation markers like NF-κB and TNF-α [147]. Paramakrishnan and his colleagues demonstrated that the activity of MMP-13 could be inhibited by β-carotene, leading to the reversal of the neuralgia effect in female zebrafish suffering from STZ-associated diabetic neuropathic pain [148]. In a study on STZ-induced diabetic rats, bixin treatment was associated with a reduction in mechanical allodynia, depressive symptoms, and anxiety-like behaviors. In addition, bixin treatment modulates oxidative parameters (GSH and lipid peroxidation) in different body regions of rats, including the hippocampus, prefrontal cortex, and lumbar spinal cord [149]. Raafat *et al.* presented evidence of the anti-inflammatory and anti-nociceptive effects of picrocrocin, crocin, and safranal, compounds found in saffron, on diabetic neuropathy [150].

#### 4.2.2 Carotenoids and Infectious Neuropathy

Infectious neuropathies are a group of conditions where nerve damage is caused by infections from various pathogens, including viruses, bacteria, fungi, and parasites [151]. Some examples of infectious neuropathies include herpes and human immunodeficiency virus (HIV) [152]. Infectious neuropathies in herpes, such as those caused by varicella-zoster virus (VZV) and herpes simplex virus (HSV), result from a combination of direct viral effects on nerve tissues, inflammatory responses, and immune-mediated processes [153]. HIV is indeed associated with distal symmetric polyneuropathy (DSP). DSP is a common neurological complication of HIV infection and is characterized by damage to the peripheral nerves, usually starting in the feet and hands and gradually progressing upwards [154].

In a study, researchers investigated the potential antiviral activity of carotenoids derived from *Kocuria* sp. RAM1 against HSV infection in Vero cells [155]. Also, *in vitro* experiments demonstrated that extracts derived from the microalgae *Dunaliella salina* and *Haematococcus pluvialis*, known for their high carotenoid content, including β-carotene and astaxanthin, effectively decreased the activity of HSV [156]. Astaxanthin has been shown to inhibit Stimulator of Interferon Genes (STING) carbonylation induced by HSV infection. This inhibition leads to enhanced antiviral responses by reducing lipid peroxidation and promoting STING translocation to the Golgi apparatus, oligomerization, and activation of the STING-dependent host defenses [157]. STING is a protein that is located in the endoplasmic reticulum membrane of cells. It acts as a sensor for the presence of viral DNA in the cytoplasm [158]. Activation of the STING pathway recruits and activates kinases like TANK-binding kinase 1 (TBK1) and IκB kinase ε (IKKε) [159], which then phosphorylate transcription factors such as interferon regulatory factor 3 (IRF3) and NF-κB. These factors move into the nucleus to stimulate the expression of genes that produce type I interferons and other antiviral molecules, playing a vital role in the immune response against viral infections [160, 161]. Zeaxanthin demonstrated strong antiviral properties against HSV, specifically by interfering with the virus’s ability to attach to and enter host cells [162]. The *in vitro* study suggests that crocin and picrocrocin from saffron have the potential to be effective anti-HSV-1 and anti-HIV agents [163].

The findings indicate that HIV-infected patients at all stages of the disease have a deficiency of β-carotene, α-carotene, and β-cryptoxanthin. These deficiencies may play a role in immune dysfunction among HIV patients [164]. In another study conducted on 182 HIV/AIDS-diagnosed adults, it was found that approximately 4% of them exhibited insufficient vitamin A levels (<0.70 μmol/L), whereas 98% had β-carotene concentrations below 1.0 μmol/L [165]. It has been observed that a deficiency in β-carotene may suggest difficulties with absorbing nutrients. However, the consumption of supplements has proven to positively affect specific T lymphocyte groups and enhance the immune system of individuals with HIV [166]. The findings of an area study indicated that infants in Uganda who had insufficient levels of plasma carotenoids were more likely to face a higher risk of mortality during HIV infection [167].

Contrary to previous studies, Sheehan found no evidence to suggest that β-carotene supplementation has any impact on the pharmacokinetics of nelfinavir or its active metabolite M8 in patients infected with HIV-1. Nelfinavir is commonly prescribed as a protease inhibitor for the management of HIV-1 infection. It is primarily metabolized by cytochrome P450 (CYP) 3A enzymes, particularly CYP3A4, and also undergoes significant metabolism to its active metabolite M8 [168].

#### 4.2.3 Carotenoids and Chemotherapy-induced Peripheral Neuropathy

Chemotherapy-induced peripheral neuropathy (CIPN) is a prevalent and debilitating consequence of cancer treatment that can greatly affect a patient's quality of life [80]. The pathophysiology of CIPN is multifactorial, and these mechanisms act in concert to cause characteristic sensory disturbances and neuropathic pain [169]. Chemotherapeutic agents significantly disrupt the cytoskeletal structure of neurons, resulting in axonal fragmentation in sensory neurons. This disruption affects the microtubule cytoskeleton and hinders the transport of mitochondria and mRNA along the axon, contributing to CIPN [170]. Moreover, exposure to these agents causes mitochondrial swelling in peripheral nerves, which triggers the release of cytochrome c and the degeneration of sensory fibers. Increased ROS levels and oxidative stress from mitochondrial dysfunction are key mechanisms [170, 171].

Additionally, these agents induce changes in the expression and function of various ion channels like potassium channels, sodium channels, and TRP channels in DRG neurons, altering their excitability and contributing to CIPN symptoms. Also, they trigger neuroinflammation by activating glial cells and altering the levels of chemokines in DRG neurons. Other mechanisms include DNA damage, myelin sheath damage, and apoptosis [172, 173].

A study reviewed that lycopene has been proven to inhibit the growth and proliferation of prostate cancer cells effectively, induce cell cycle arrest, and promote apoptosis both in *in vivo* and *in vitro* studies [174]. However, according to the findings of Greenlee *et al.,* there was an increased risk of developing CIPN in women who began taking antioxidants such as β-carotene, selenium, vitamin C, vitamin E, and zinc alongside their chemotherapy treatment. Additionally, these effects were found to endure for a duration of up to two years [175].

**Fig. (4):**
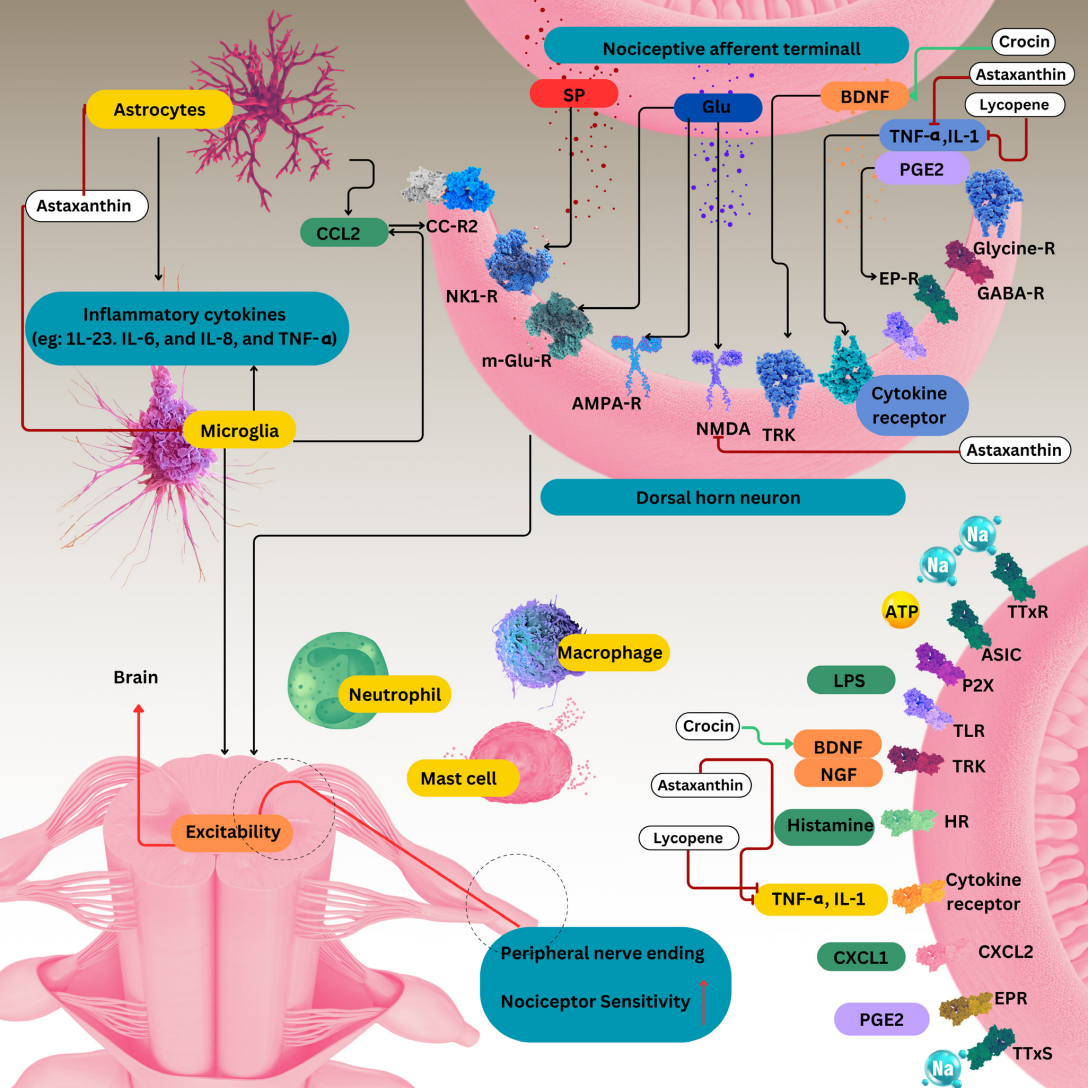
Molecular pathways mediating carotenoid effects on peripheral neuropathic pain. **Abbreviations:** Acid-sensing ion channel (ASIC), Adenosine triphosphate (ATP), Alpha-amino-3-hydroxy-5-methyl-4-isoxazolepropionic acid receptor (AMPA-R), Brain-derived neurotrophic factor (BDNF), chemokine (C-C motif), ligand 2 (CCL2), CXC chemokine receptor (CXCR), Prostaglandin E receptor (EP-R), Gamma-aminobutyric acid receptor (GABA-R), Glutamate (Glu), Interleukin (IL), Lipopolysaccharide (LPS), Metabotropic glutamate receptor (m-Glu-R), Nerve growth factor (NGF), Neurokinin 1 Receptor (NK1-R), N-methyl-D-aspartate (NMDA), Prostaglandin E2 (PGE2), substance P (SP), Tetrodotoxin receptor (TTxR), Tetrodotoxin-sensitive (TTXS), Toll-like receptor (TLR), Tropomyosin receptor kinase (TRK), Tumor necrosis factor-alpha (TNF-α).

Overall, while there is positive evidence from *in vitro* and *in vivo* studies on animal models regarding the analgesic effects of carotenoids, it remains unclear whether carotenoids can be definitively considered useful for reducing neuropathic pain in cancer patients (Fig. **4**).

### 4.3 Carotenoids and Inflammatory Pain

Inflammatory pain is a protective response to tissue damage or infection, marked by increased sensitivity to harmful stimuli. This type of pain arises from the action of various pro-inflammatory substances released during the inflammatory response, leading to both acute and chronic pain conditions [176].

Key players in the development of inflammatory pain include pro-inflammatory mediators such as cytokines, prostaglandins, bradykinin, and substance P [177]. These substances are released by immune cells, damaged tissues, and blood vessels in response to injury or infection [178]. Prostaglandins are particularly significant as they sensitize nociceptors—pain-sensing nerve fibers—by lowering their activation thresholds, which heightens pain perception. Nociceptors can respond to a range of stimuli, including mechanical, thermal, and chemical signals associated with inflammation. The release of inflammatory mediators enhances the sensitivity of these receptors, making them more reactive to pain signals [176, 179].

Additionally, immune cells such as neutrophils and macrophages infiltrate inflamed tissues, releasing further inflammatory mediators that perpetuate the cycle of inflammation and pain. This infiltration not only sustains ongoing inflammation but also amplifies the release of substances that sensitize nociceptors [6]. The overproduction of ROS during inflammation activates pro-inflammatory pathways, resulting in the release of cytokines. This oxidative stress contributes to neuronal sensitization and the development of chronic pain states. ROS can also impair mitochondrial function in neurons, leading to energy deficits that exacerbate pain signaling pathways. Chronic inflammation can induce central sensitization [36, 180]. The intricate interplay among inflammatory mediators, nociceptor sensitization, immune cell activity, oxidative stress, and central sensitization form a complex network that underlies inflammatory pain. A thorough understanding of these mechanisms is essential for developing effective treatments aimed at alleviating both acute and chronic inflammatory pain conditions.

Animal models of inflammatory pain often utilize substances like complete Freund’s adjuvant (CFA), formalin, carrageenan, capsaicin, and lipopolysaccharide (LPS) to simulate tissue injuries. In addition to these models, researchers have also created models of pain hypersensitivity by inducing injuries through burning, freezing, and ultraviolet irradiation. [181, 182]. CFA activates NF-κB and MAPK pathways, releasing pro-inflammatory cytokines that contribute to hyperalgesia by sensitizing neurons [183]. Formalin induces a biphasic pain response, with an acute phase from direct nociceptive stimulation and a prolonged phase driven by inflammatory mediators [184]. Carrageenan enhances sensory neuron excitability and causes allodynia through increased TRPV1 expression, while capsaicin initially induces pain but leads to neuronal desensitization and analgesia in inflamed tissues [185]. LPS triggers an immune response that releases inflammatory mediators, activating nociceptors and contributing to hyperalgesia *via* similar pathways as CFA [186]. Together, these models help researchers understand persistent pain mechanisms and explore potential therapeutic interventions (Table **3**).

#### 4.3.1 Carotenoids and Complete Freund’s Adjuvant (CFA)-induced Inflammatory Pain

The CFA-induced arthritic rat model is a well-established method for studying rheumatoid arthritis and evaluating potential treatments. CFA is composed of heat-killed mycobacterium cells suspended in mineral oil and is injected into the rat’s hind paw to induce a chronic, polyarthritic condition [187]. Notable characteristics of this arthritis model include the development of mechanical allodynia, thermal hyperalgesia, and spontaneous pain behaviors within several days after CFA injection. Additionally, the injected joint experiences significant swelling and inflammation while histological changes occur in the joints. The model also exhibits systemic effects such as anemia, increased white blood cell count, and alterations in lipid profile [188].

The oral administration of astaxanthin demonstrated remarkable efficacy in reducing arthritis symptoms. It enhanced pain tolerance, reduced paw edema, and improved arthritis scores. In addition, astaxanthin treatment has significantly suppressed the levels of inflammatory and oxidative mediators, such as TNF-α, C-reactive protein (CRP), cyclic citrullinated peptide (CCP) antibodies, and MDA in the rats administered with CFA [189]. Zhao *et al.* showed that the ability of astaxanthin to reduce inflammatory pain in the CFA model is related to the regulation of MAPK and Nrf2/HO-1 p38 pathways [190]. The inflammatory hyperalgesia and hyperexcitability of trigeminal nociceptive neurons caused by CFA injection into the rats’ whisker pads were significantly reduced when lutein was administered at 10 mg/Kg. This was achieved through the inhibition of the peripheral Cox-2 signaling cascade [191]. In a study using a rat model of chronic prostatitis/chronic pelvic pain syndrome (CP/CPPS) induced by complete Freund’s adjuvant, lycopene administration exhibited significant benefits. It effectively mitigated the levels of chemokines MCP1 and MIP-1α, decreased the expression of pro-inflammatory cytokines such as TNFα and ILs, enhanced antioxidant enzyme activity, including CAT, GSH-PX, and T-SOD, and resulted in a reduction in MDA levels [192]. The administration of crocin, either alone or in combination with curcumin, resulted in a reduction in paw swelling and a decrease in liver and serum markers, as well as inflammatory cytokines IL-lβ and TNF-α, in rats with a CFA model [193]. Both iridoid glycosides and crocetin derivatives extracted from Gardenia jasminoides Ellis demonstrated significant anti-arthritic effects in a mouse model of FAC-induced arthritis. This effect was attributed to a significant reduction in serum levels of TNF-α and transforming growth factor beta 1 (TGF-β1) [194]. The saffron metabolite, transcrocetin meglumine salt, effectively alleviated mechanical allodynia and thermal hypersensitivity in CFA-injected mice. It also inhibited microglia and astrocyte activation in the spinal cord and suppressed the production of proinflammatory cytokines like IL-1β, TNF-α, and IL-6 [195]. Oral administration of 200 mg/kg of safranal resulted in a decrease in alanine and aspartate aminotransferase, C-reactive protein levels, urea, creatinine, and MDA, COX-2, NF-κB, and TNF-α as well as increasing GPx, following the CFA model [196].

#### 4.3.2 Carotenoids and Formalin-induced Inflammatory Pain

The formalin test is a commonly used model for rodents' pain and nociception. It includes injecting a diluted formaldehyde (formalin) solution subcutaneously or intradermally into the animal's hind paw or other body areas. This test shows two specific phases of nociceptive behavior: an initial acute phase lasting around 5 minutes post-injection, likely resulting from direct nociceptor stimulation, and a subsequent late tonic phase emerging 20-30 minutes post-injection, believed to trigger an inflammatory response [197]. The effectiveness of carotenoids in reducing this inflammatory response has been investigated in several studies. In previous research, we discovered that the pain-relieving effects of astaxanthin after the formalin test can be attributed to activating the l-arginine/NO/cGMP/KATP pathway [198]. *Meso-zeaxanthin* administration resulted in a noteworthy reduction in paw edema induced by formalin, carrageenan, and dextran in mice. Moreover, it exhibited a significant anti-inflammatory effect in lipopolysaccharide (LPS)-stimulated macrophages by effectively decreasing the production of iNOS, CRP, TNF-α, COX-2, IL-1β, and IL-6 [199]. Oral treatment with bixin (15 or 30 mg/kg) significantly reduced formalin-induced flinches in rats during both phases of the formalin test, indicating an antinociceptive effect [200]. Crocin increased the analgesic effects of morphine following the formalin test in a dose-dependent manner [201].

#### 4.3.3 Carotenoids and Carrageenan-induced Inflammatory Pain

Carrageenan, extracted from Irish moss (*Chondrus crispus*), is a sulphated mucopolysaccharide. When injected, it primarily triggers local inflammatory responses by causing macrophages to aggregate. The inflammation caused by carrageenan is acute, does not activate the adaptive immune system, and can be consistently replicated. This has made it a useful tool for inducing hyperalgesic conditions to evaluate the efficacy of specific pain relievers [202]. A study attributed the presence of various chemicals like β-carotene, lycopene, flavonoids, phytol, and other compounds in *Wendlandia heynei* was found to be responsible for its anti-inflammatory effects in the carrageenan and formalin model. These chemicals may potentially inhibit the NF-κB pathway and other inflammatory mediators, thereby contributing to the observed anti-inflammatory activity [203]. Research indicates that Astaxanthin can attenuate paw edema, hyperalgesia, myeloperoxidase (MPO) accumulation, and MDA and superoxide anion levels induced by Carrageenan [204]. Doses 25 and 50 mg/kg of lycopene showed local anti-inflammatory effects in the carrageenan-induced paw swelling model [205]. Several studies concluded that picrocrocin, crocin, and safranal exhibit anti-inflammatory and antinociceptive effects post-carrageenan injection [150, 206]. Crocin pretreatment reduced the hind paw edema and PGE2 levels in rats' hind paws injected with carrageenan in a dose-dependent manner [207].

#### 4.3.4 Carotenoids and Capsaicin-induced Inflammatory Pain

Capsaicin, the primary spicy component in chili peppers, triggers a burning sensation, pain, and inflammatory reaction when applied to the skin or mucous membranes by activating the TRPV1 receptor on pain-sensing nerve fibers. However, with repeated or prolonged exposure to capsaicin, the sensory neurons become desensitized and unresponsive, leading to a loss of pain sensation and anti-nociceptive effects [208]. Crocin injection into the fourth ventricle of the brain effectively alleviated orofacial pain induced by capsaicin in rats [209]. Supplementation with selenium (2 mg/kg), lycopene (2 mg/kg), or zinc (10 mg/kg), alone or combined, was found to decrease capsaicin-induced mutagenicity and oxidative damage in mice. These compounds have been demonstrated to lower MDA levels, micronuclei formation, and DNA fragmentation [210].

#### 4.3.5 Carotenoids and Lipopolysaccharide-induced Inflammatory Pain

**Fig. (5):**
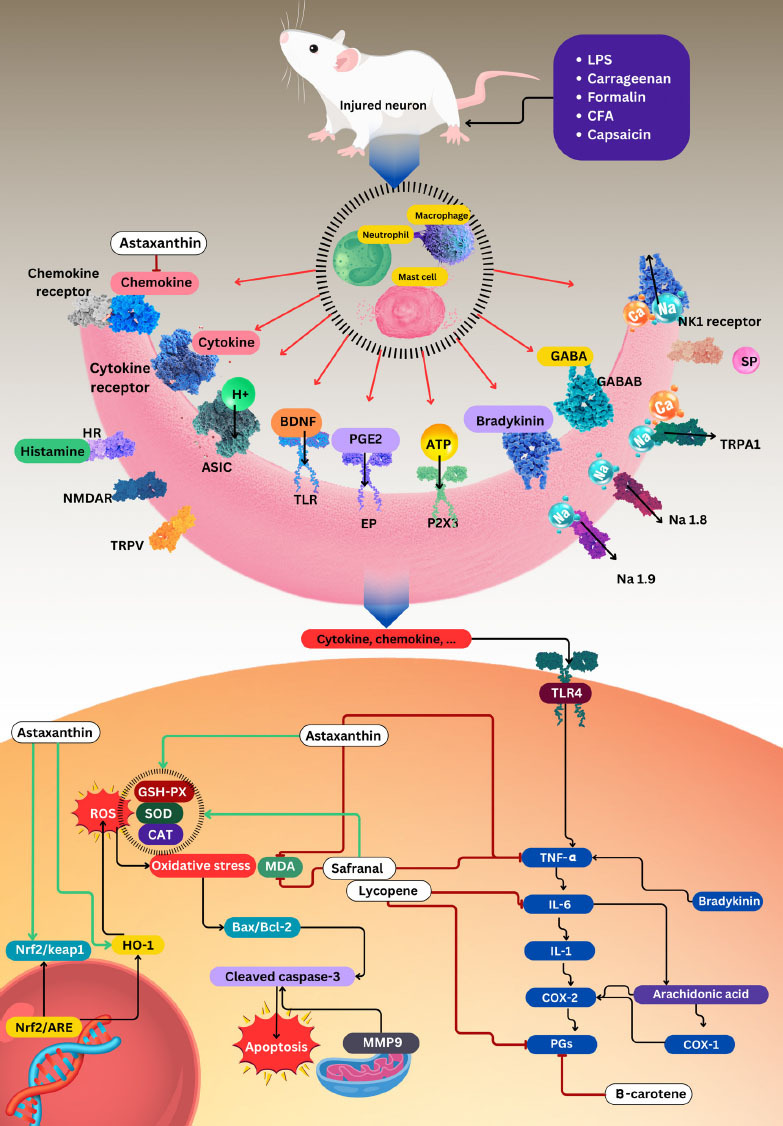
Molecular pathways mediating carotenoid effects on inflammatory pain. **Abbreviations:** Acid-sensing ion channel (ASIC), Brain-derived neurotrophic factor (BDNF), Cyclooxygenase (COX), Catalase (CAT), Complete Freund’s adjuvant (CFA), Glutathione (GSH), Glutathione-S-Transferase (GST), Gamma-aminobutyric acid receptor (GABA-R), Glutamate (Glu), Heme oxygenase-1 (HO-1), Interleukin (IL), Kelch-like ECH-associated protein 1 (Keap1), Lipopolysaccharide (LPS), Matrix metalloproteinase (MMP), Malondialdehyde (MDA), Nuclear factor erythroid 2-related factor 2 (Nrf2), Neurokinin 1 Receptor (NK1-R), N-methyl-D-aspartate (NMDA), Prostaglandin E2 (PGE2), substance P (SP), Toll-like receptor (TLR), Reactive oxygen species (ROS), Superoxide dismutase (SOD), Tropomyosin receptor kinase (TRK), Tumor necrosis factor-alpha (TNF-α), Transient receptor potential vanilloid (TRPV), TRP ankyrin member 1 (TRPA1).

LPS is a potent activator of the innate immune system and plays a key role in the recruitment and activation of inflammatory cells, contributing to pain and inflammation [211]. LPS is recognized by the TLR4 on the surface of immune cells like monocytes and macrophages. The binding of LPS to TLR4 activates downstream signaling pathways, including the NF-κB and MAPK pathways. This leads to the production and release of pro-inflammatory cytokines like TNF-α and ILs [212, 213]. These cytokines then prompt the production of hyperalgesia mediators like prostaglandins and sympathomimetic amines, which contribute to the inflammatory pain response [214]. These inflammatory mediators can sensitize peripheral nociceptors, leading to increased excitability and responsiveness to noxious stimuli [215]. Additionally, they can activate immune cells in the surrounding tissue, leading to the recruitment and activation of additional inflammatory cells, amplifying the inflammatory response, and contributing to the development and maintenance of pain [216, 217]. FLexPro MD is a combination of krill oil, astaxanthin, and hyaluronic acid that aims to alleviate knee joint pain in humans. This combination significantly inhibited the mRNA levels of IL-6, TNF-α, and IL-1β and phosphorylation of NF-κB p65 and IκB-α, as well as iNOS, MMPs, and COX-2 in LPS induced inflammatory in *in vitro* and *in vivo* pain models (Fig. **5**) [218].

## 5 CLINICAL STUDIES OF THE EFFECTIVENESS OF CAROTENOIDS IN PAIN MANAGEMENT

Although carotenoids show promise in treating neuropathic and inflammatory pain in animals, further well-designed clinical trials are essential to confirm their effectiveness and safety in human pain management. The existing evidence remains limited and preliminary, highlighting the need for more robust research in this area. Recently, a clinical study revealed that administering a daily dose of crocin (30 mg) twice a day for 8 weeks may result in a decrease in inflammation markers (high-sensitivity C-reactive protein) among patients with MS [219]. Also, the therapeutic effects of crocin in patients with MS have been reported to reduce inflammation, specifically targeting TNF-α and IL-17 levels, decrease oxidative stress such as MDA, and alleviate DNA damage [220]. The administration of crocin-selenium nanoparticles for 12 weeks improved cognitive function and modulated oxidative stress markers (TAC, MDA) in MS patients [221].

Furthermore, supplementing with 20 mg/day of lutein for 12 weeks improved attention and information processing speed and increased carotenoid levels in individuals with MS [222]. In a randomized, double-blind, placebo-controlled trial involving patients with CIPN, the administration of crocin for 8 weeks demonstrated a significant reduction in sensory, motor, and neuropathic pain [223]. Adding lycopene to orchidectomy not only shrinks the primary tumor but also reduces secondary tumors and relieves bone pain and lower urinary tract symptoms. Lycopene has demonstrated a strong ability to inhibit the growth and proliferation of prostate cancer cells, induce cell cycle arrest, and trigger apoptosis, both *in vivo* and *in vitro* [224]. A prospective study involving 20 patients with metastatic hormone-refractory prostate cancer also confirmed the analgesic effects of lycopene [225].

## CONCLUSION

In conclusion, this review highlights the significant potential of carotenoids as therapeutic approaches for managing pathological pain, particularly neuropathic and inflammatory pain. These pain types share significant commonalities, such as the activation of nociceptive pathways, neuroinflammation, and central sensitization. Chronic pain conditions often involve altered pain processing and are influenced by factors like oxidative stress and psychosocial elements. Acknowledging these similarities is crucial for developing effective therapeutic approaches that can address the diverse manifestations of pathological pain, ultimately enhancing patient outcomes and quality of life. Also, the intricate interplay of various biological pathways, including neuroinflammation, oxidative stress, and changes in neurotransmitter signaling, underscores the need for multifaceted treatment strategies. Carotenoids, a group of naturally occurring pigments found in various fruits and vegetables, exhibit notable anti-inflammatory and antioxidant properties. Their ability to modulate several mechanisms involved in pain perception/modulation positions them as promising candidates for pain management. These compounds demonstrate the potential to reduce pain sensitivity and influence key signaling pathways related to pain processing, including the modulation of pro-inflammatory mediators and the activation of antioxidant responses. Despite the encouraging findings, the current body of evidence-primarily stems from animal studies, and further well-designed clinical trials are essential to validate the safety and efficacy of carotenoids in human populations. Future research should focus on elucidating optimal dosages, assessing long-term effects, and exploring potential interactions with conventional pain therapies. Additionally, understanding the safety profile of carotenoids when used as supplements, especially in vulnerable populations, is crucial. While carotenoids have shown promising effects in combating different pain types, some pharmacokinetic limitations urge the need for providing novel formulations like polymeric/metallic nanoparticles, micelle, solid-lipid nanoparticles, and liposome [226].

In summary, the multifaceted biological actions of carotenoids, coupled with their natural origins and safety profiles, position them as attractive candidates for innovative pain management solutions. Continued research is warranted to explore their full therapeutic potential, optimize formulations, and integrate these compounds into comprehensive pain management strategies, ultimately improving the quality of life for individuals suffering from chronic pain.

## STUDY LIMITATIONS

While this review highlights the therapeutic potential of carotenoids in managing neuropathic and inflammatory pain, some limitations should be acknowledged. First, much of the current evidence is derived from preclinical studies conducted in animal models. Although these findings provide valuable mechanistic insights, they may not fully replicate the complexity of human pain conditions. Second, clinical data on carotenoids in pain management remain limited, with small sample sizes, heterogeneous study designs, and inconsistent dosing regimens, making it difficult to draw definitive conclusions. Third, variability in carotenoid bioavailability, influenced by dietary sources, metabolism, and genetic factors, poses challenges for standardizing the therapeutic use of carotenoids.

Additionally, potential adverse effects associated with excessive supplementation, particularly in vulnerable populations such as smokers, require careful consideration. Finally, this review does not cover possible synergistic interactions with other nutraceuticals or pharmacological agents, which may further influence therapeutic efficacy. Future well-designed clinical trials are essential to validate the analgesic effects of carotenoids, establish optimal dosing strategies, and ensure their safety and efficacy in diverse patient populations.

## Figures and Tables

**Table 1:** Carotenoids and derived compounds in central neuropathic pain.

**Disease**	**Treatment**	**Induction Method**	**Animal/Dose**	**Effects**	**References**
SCI	Astaxanthin	Compression	Male rats 10 μl of 0.2 mM, IT, 30 minutes after surgery	↓ NR2B, p-p38MAPK, TNF-α, HMGB1, TLR4, NF-κB, GFAP, MMP-9, AQP4, MDA, P62, Bax, p-ERK/ERK, MIF ↑ TAC, SOD, GSH-PX, Bcl2, LC3B, Beclin1, p-AKT/AKT, Doublecortin, PGC1α, NRF1, TFAM	[43, 45-48, 108]
			Female rats 80 mg/kg, IP, 5 minutes after surgery	↓ NT-3	[50]
		Contusion	Male rats 10 μl of 0.2 mM, IT, 30 minutes after surgery	↓ COX-2, TNF-α, IL-1β, IL-6, Bax, Caspase-3, MDA ↑ Bcl2, CAT, SOD, GSH-PX	[42, 44]
			Male and female rats 75 mg/kg, PO, Twice per day	↓ MDA, Apoptosis index ↑ SOD	[49]
	Lutein	Spinal cord ischemia-reperfusion injury	Male rats 0.2, 0.4 mg/kg, IP, 30 minutes before surgery	↓ MDA ↑ TAC	[56]
	Crocin	Contusion	Female rats 50 mg/kg, IM, Twice a day, for 3 days	↓ CGRP	[57]
	β-Carotene	Compression	Male rats 10, 20, 40, 80 mg/kg, IP, Immediately after the surgery	↓ ROS, MDA, NO, GFAP, Nuclear p65, nuclear p- IкBα, TNF-α, IL-1β, IL-18, COX-2 ↑ SOD, Nrf2, HO-1, Cytosolic p65, IкBα	[52]
	Lycopene	Contusion	Male C57BL mice 4 mg/kg, Immediately after surgery	↓ TNF-α, NF-kB ↑ ZO-1, Claudin-5, HO-1	[54]
			Male rats 5, 10, or 20 mg/kg, IP	↓ Caspase-9, Caspase-3, Bax, Cytosolic Cyt C, MDA ↑ Mitochondrial Cyt C, Bcl2, Cyt b, TFAM, SOD, GSH-Px	[55]
		Spinal cord ischemia-reperfusion injury	Rats 25, 50 mg/kg, PO, 2 weeks	↓ Apoptosis index, TNF-α, IL-1β, IL-6, IL-8, COX-2, NF-κB, AP-1	[53]
Stroke	Astaxanthin	MCAO Cortical neurons exposed to H_2_O_2_	Male rats 20, 50, 80 mg/kg, PO, Twice at 5 hours and 1 hour before ischemia 250, 500, 1000, and 2000 nM	↓ Apoptotic cells ↑ MMP	[61]
		Ischemic-Hypoxic Reperfusion OGDre	Male C57BL/6J mice pups P7 mice pups 40, 80 mg/kg bEnd.3 cells 20 μM	↓ p75^NTR^, ROS ↑ ZO-1, HIF-1α, claudin-5	[67]
		MCAO	Male rats 5, 10 mg/kg, PO, 1 week before ischemia	↓ MDA, Bax, GFAP, MAP-2 ↑ SOD, Nrf2, HO-1, NQO1, Bcl2, BDNF, GAP-43	[62]
			Male rats 25, 45, 65 mg/kg	↓ NF-kB, TOS, TNF-α, P53, PUMA, Caspase-3 ↑ GSH-PX, GLT-1, CAT	[63]
			Male rats 20, 40, 80 mg/kg, PO, 1 week	↓ MDA, ↑ BDNF, NGF, CAT, SOD, GSH-Px	[64]
			Rats 100 mg/kg, PO, 3 days before ischemia	↓ MDA, TNF-α, IL-1β, IL-6, MDA, Bax, Cytosolic Nrf2 ↑ Bcl2, SOD, CAT, GSH-PX, Nuclear Nrf2	[65]
			20 μl of 0.1 mM ICV, 15 minutes before ischemia	↓ MDA, Glutamate release ↑ Aconitase, Cyt C	[66]
			Male C57BL/6 mice 30 mg/kg, PO, Twice a day for 4 weeks	↑ GAP43, cAMP, PKA, p-CREB, PKAc	[68]
	Lutein	MCAO	Male C57BL/6N mice 0.2 mg/kg, IP, 1 hour after ischemia	↓ NT, PAR, NF-kB, p-ERK/ERK, p- IκB/IκB, Cox-2 p-AKT/AKT, Bcl-2, Hsp-70	[69]
Stroke	β-carotene	SHRSP	Primary astrocytes from the cerebral cortices of female WKY/Izm and SHRSP/Izm rats 30 μM, 2, 4, 8 hours	↑ ApoE, Abca1, Abcg1, Hmgcr, Fgf1, Fgfr1	[70]
	Lycopene	MCAO	Male rats 5, 20 mg/kg, PO, 15 days before ischemia	↓ LD, NOS, ROS, ↑ HIF-1α, Bcl2	[71]
			Male rats 6 mg, 2 weeks	↓ Caspase-3, p-JNK/JNK, MDA, NO, iNOS, nNOS, HO-1, NOX-2, L-Ferritin, IL-6, p-STAT3/STAT3 ↑ Bcl2, SOD, GSH, CAT, FPN1	[72]
	Crocin	ICH	Male C57B/L mice 40 mg/kg, PO, 6 hours after ICH and then every 12 hours for up to 7 days	↓ ROS, Number of iron positive cells, HO-1	[73]
			40 mg/kg	↓ MDA, Cytosolic Nrf2 ↑ Nuclear Nrf2, GSH-PX, SOD, *FTH1, GPX4, SLC7A11*	[74]
		MCAO OGD/R	Male rats 5, 100 mg/kg, PO, 2 weeks before ischemia HT22 cells 25, 50, 100 μM	↓ LC3, ULK1, p-AMPK/AMPK ↑ p62, p-mTOR/ mTOR	[75]
		MCAO	Male rats 10, 20, 40, or 60 mg/kg, PO, Every two days for 8 weeks before ischemia	↓ MMP-2, MMP-9, NOX ↑ ZO-1, Occludin, Claudin-5	[76]
			Male rats 15, 30, 60, 120 mg/kg, IP	↓ MDA ↑ SOD, GSH-PX, TAC	[77]
			Male rats 50, 80 mg/kg, IP, At the beginning of ischemia	↓ Number of eosinophilic neurons at ischemic area	[79]
		MCAO Serum-deprived and hypoxic	Male ddY mice 10 mg/kg, IV, immediately before and 3 hours after ischemia PC12 cell 10 μM	↓ Caspase-3 ↑ γ-GCS, GSH	[78]
		Global cerebral ischemia-reperfusion	Female rats 40 mg/kg, PO, 10 days	↓ Number of TUNEL positive cells, OSI, Caspase-3, HIF-1α ↑ TAS, TOS	[80]
	Safranal	Global cerebral ischemia-reperfusion	Male NMRI rats 727.5, 363.75, 145.5, 72.75 mg/kg, IP 5 minutes before ischemia and every 24 hours for 3 days after ischemia	↓ MDA ↑ Antioxidant capacity	[81]
		MCAO	Male rats 72.5, 145 mg/kg, IP, within 0, 3, and 6 hours after ischemia	↓ MDA ↑ Antioxidant capacity, Total thiol	[82]
MS	Astaxanthin	Cuprizone	Male rats 3 mg/kg, 4 weeks	↑ MBP, MOG, PDGFR-α	[88]
		EAE	Female C57BL/6 mice 400 mg/kg 3 weeks	↓ IL-1β, IL-6	[89]
	Crocin	EAE	Female C57BL/6 mice Male CHOP2/2 mice Mature oligodendrocytes from rat 100 mg/kg, IP, 100, 200, 400 μM	↓ Syncytin-1, BiP, XBP1/s, IFNa, Mx1, NOS2, HIF-1a, CHOP, CD3ɛ, F4/80, TNF-α ↑ CNPase, CD3^+^, MOG	[90]
		Cuprizone	Male C57BL/6 mice 100 mg/kg, PO, 3 times per week for 5 weeks	↓ MDA ↑ GSH-PX, SOD, TAS	[91]
	Bixin	EAE	Female C57BL/6 mice 50, 100, 200 mg/kg, PO From the 3^rd^ day to the 15^th^ day	↓ 3-NT, IL-1β, IL-18, TXNIP, NLRP3, TNF-α, IL-6, IL-8 ↑ Nrf2, CAT, NQO-1, SOD2, IL-10	[92]
TBI	β-carotene	Weight drop	Male C57BL/6 mice 10, 20, 30, 50 mg/kg, PO 3 hours after TBI, and then every day afterwards.	↓ MDA, Bax, Caspase-3, Cytosolic Nrf2, Keap1, NQO1, HO-1 ↑ SOD, Nuclear Nrf2, Bcl2	[105]
	Astaxanthin	Weight drop	Male C57BL/6 mice Nrf2 gene knockout mice Primary cortical neurons 25, 75, 150 mg/kg 25, 50, 100 μM PO, 3 hours after TBI, and then every day afterwards.	↓ p-p38, Bax, Caspase-3, Cytosolic Nrf2, 8-OHG ↑ SIRT1, Nuclear Nrf2, Prx2, ASK1, Bcl2	[98]
			Male ICR mice 25, 75 mg/kg, PO, 1 week	↑ BDNF, GAP-43, SYP, Synapsin	[99]
		Controlled cortical impact	Male C57BL/6N mice 100 mg/kg, IP, 30 minutes after TBI	↓ Cleaved-Caspase-3 Nrf2, NQO1, HO-1, SOD1	[101]
			Male C57BL/6 mice 10, 25, 50, 100 mg/kg, IP, 30 minutes after TBI	↓ AQP4, NKCC1, GFAP	[102]
	Lutein/ Zeaxanthin	Cold injury-induced trauma	Male C57BL/6j mice 20 mg/kg 1 week after TBI	↓ IL-1β, IL-6, NF-κB, ↑ BDNF, GAP-43, ICAM, NCAM	[103]
	Crocetin	Weight drop	Male C57BL/6j mice 50 mg/kg, PO, 3 days	↓ TNF-α, IL-1β, IL-6, NF-κB, p62, Caspase-3 ↑ LC3II/I, Beclin-1	[106]
	Crocin	Weight drop	Male albino BABL/c mice 30 mg/kg, IP, 30 minutes before TBI	↓ MPO, MDA, TNF-α, IFN-γ ↑ GSH	[107]

**Abbreviations**: 3-nitrotyrosine (3-NT), 8-hydroxyguanosine (*8*-*OHG*), Activator protein 1 (AP-1), Apolipoprotein E (APOE), Apoptosis signal-regulating kinase 1 (*ASK1*), Aquaporin-4 (AQP4), B-cell lymphoma 2 (Bcl2), Bcl2-associated X (Bax), Brain derived neurotrophic factor (BDNF), Calcitonin-gene related peptide (CGRP), cAMP-dependent protein kinase (PKAc), cAMP-response element binding protein (CREB), Catalase (CAT), Cyclooxygenase-2 (COX-2), Cytochrome C (Cyt C), Experimental autoimmune encephalomyelitis (EAE), Extracellular signal-regulated kinase (ERK), Ferritin heavy chain 1 (FTH1), Ferroportin (FPN1), Fibroblast growth factor 1 (FGF1), Glial fibrillary acidic protein (GFAP), Glutamate transporter 1 (GLT-1), Glutathione peroxidase (GSH-PX), Growth-associated protein-43 (GAP-43), Heme-oxygenase 1 (HO-1), High density lipoproteins (HDL), High mobility group box 1 (HMGB1), Interleukins (ILs), Intracellular adhesion molecule (ICAM), Intracerebral hemorrhage (ICH), Intramuscular (IM), Intraperitoneal (IP), Intrathecal (IT), Intravenous (IV), JAK (Janus Kinase), Lactic acid (LD), Light chain 3 (LC3), Macrophage migration inhibitory factor (MIF), Malondialdehyde (MDA), Matrix metallopeptidase- 9 (MMP-9), Microtubule-associated protein2 (MAP-2), Middle cerebral artery occlusion (MCAO), Mitochondrial membrane potential (MMP), Mitochondrial transcription factors A (TFAM), Mitogen-activated protein kinases (MAPKs), Monocyte chemotactic protein 1(MCP-1), Multiple sclerosis (MS), Myelin basic protein (MBP), Myelin oligodendrocyte protein (MOG), Myeloperoxidase activity (MPO), NAD(P)H quinone oxidoreductase 1 (NQO1), Na–K–Cl cotransporter (NKCC), Nerve growth factor (NGF), Neural cell adhesion molecule (NCAM), Neurotrophin-3 (NT-3), Nicotinamide adenine dinucleotide phosphate oxidase 2 (Nox2), Nitric oxide synthetase (NOS), Nitric-oxide (NO), Nitrotyrosine (NT), NLR family pyrin domain containing 3 (*NLRP3*), N-methyl D-aspartate receptor subtype 2B (NR2B), Nuclear factor-kappa B (NF-κB), Nuclear respiratory factor (NRF), Oral gavage (PO), Oxidative stress index (OSI), Oxygen glucose deprivation/re-oxygenation (OGD/R), Oxygen-glucose deprivation/reperfusion (OGDre), P53 upregulated modulator of apoptosis (PUMA), Peroxiredoxin 2 (Prx2), Peroxisome proliferator-activated receptor-γ coactivator (PGC1α), Platelet-derived growth factor-alpha (PDGFR-α), Poly ADP-ribose (PAR), Protein Kinase A (PKA), Reactive oxygen species (ROS), Signal transducer and activator of transcription 3 (STAT3), Stroke-prone spontaneously hypertensive rat (SHRSP), Superoxide dismutase (SOD), Spinal cord injury (SCI), Synaptophysin (SYP), *Thioredoxin-interacting protein* (*TXNIP*), Traumatic brain injury (TBI), Toll-like receptor 4 (TLR4), Total antioxidant capacity (TAC), Total antioxidant status (TAS), Total oxidant status (TOS), Tumor necrosis factor alpha (TNF-α), Unc51-like autophagy activating kinase 1 (*ULK1*), Zonula occludens 1 (ZO)-1, γ-glutamylcysteine synthetase (*γ*-*GCS*).

**Table 2:** Carotenoids and derived compounds in peripheral neuropathic pain.

**Treatment**	**Induction Method**	**Animal/Dose**	**Effects**	**References**
Astaxanthin	CCI	Male rats, 1 μg, IT, 10 days	↓TNF-α, IL-1β ↑ SOD, GSH-PX, HO-1	[125]
		Male albino Swiss mice, 0.5, 1, 2 µg/ 5 µL, IT, 1 week after CCI	Astaxanthin (MAPK inhibitor, Nrf2 activator), apart from its pain-relieving properties, also effectively enhances opioid analgesia in neuropathy	[126]
Trans-astaxanthin		Male ICR mice, 10, 40, 80 mg/kg, PO, twice per day, began 1 week after CCI 3 weeks	↓TNF-α, IL-1β, IL-6, IDO1	[127]
Crocin		Male rats, 15, 30, 60 mg/kg, IP, 2 weeks 6 µg/5 µL, ICV	↓Thermal hyperalgesia and mechanical allodynia, BDNF	[128, 130-132]
		Male rats, ICV	Effects of crocin on pain/anxiety responses and synaptic plasticity mediated by central α2-adrenoceptor.	[133]
Astaxanthin	SNL BV2 microglial cell PC12 cells	Male C57BL/6 mice, 5, 10 mg/kg, IP, 5 days after SNL until day 28 5, 10 μM	↓ Iba-1, GFAP, C-fos, TNF-α, IL-1β, IL-6, IL-17, p-ERK/ERK, p-p38, NF-κB ↑ IL-4, IL-10, NF-κB p65	[134]
Lycopene	PSNL Spinal astrocytes	Male ddy mice, 10 nmol/5 μ, IT, Four times (7, 9, 11, and 13 days after PSNL) 5, 20 μM	↑ Cx43	[135]
	PSNL	Male rats, 25, 50 mg/kg, IP, 2 weeks	↓ LPO ↑ CAT, GSH, SOD	[136]
β-carotene	Diabetic neuropathy STZ	Female zebrafish 25, 50, 100 µM, Day 11 to 22	↓ TBARS, MMP-13 ↑ GSH	[148]
Lycopene	Diabetic neuropathy STZ	Male rats, 1, 2, 4 mg/kg, PO, 4 weeks	↓ TNF-α, NO	[145, 146]
	Diabetic optic neuropathy Alloxan	Albino male rats, 4 mg/kg, PO, 12 weeks	↓ TNF-α, NF-κB, OSI, TOS, MDA ↑ GSH, TAS	[147]
Bixin	Diabetic neuropathy STZ	Male rats, 10, 30, 90 mg/kg, PO, Twice a day, Day 14 to 31	↓ % Glycated hemoglobin, LPO ↑ GSH	[149]
Safranal	Diabetic neuropathy Alloxan	Male albino mice, 15, 20, 25 mg/kg	↓ Blood glucose, HbA1c, TBARS ↑ Serum insulin, GSH, CAT	[150]
Astaxanthin	HSV-1 infection	C57BL/6J mice Sting-deficient mice 25 mg/kg, 1 week Mouse primary peritoneal macrophages Human embryonic kidney cells Mouse embryonic fibroblasts 0 to 160 mM, 1 hour	Inhibit HSV-1 infection ↓ STING carbonylation, MDA ↑*Mx1, IFIT1, IFIT2, ISG15,* and *CXCL10, IFN-b*	[157]
Zeaxanthin	HSV-1 infection	Vero cells 0.4, 0.6, 0.8, 1.2, 1.6 mg/mL, 72 hours	Inhibits the HSV-1 replication acting ↓ Viral plaques	[162]
Carotenoids	HSV-1 infection	Vero cells 100, 200, 250 µg/ml	Inhibit HSV-1 infection	[155, 156]
Saffron Crocin Picrocrocin	HSV-1 infection HIV-1 infection	Human embryonic kidney cells African green monkey kidney cells Human cervical cancer cells (Hela) saffron (0.1-8 mg/ml), crocin (0.5-5 mM), picrocrocin (0.5-10 mM), 72 hours	Anti-HSV-1 and Anti-HIV-1 activity	[163]

**Abbreviations**: Brain derived neurotrophic factor (BDNF), Catalase (CAT), Chronic constriction injury (CCI), Chemotherapy-induced peripheral neuropathy (CIPN), Connexin 43 (Cx43), Extracellular signal-regulated kinase (ERK), Glial fibrillary acidic protein (GFAP), Glutathione peroxidase (GSH-PX), Heme-oxygenase 1 (HO-1), Human immunodeficiency virus (HIV), Herpes simplex virus (HSV), Ionized calcium-binding adapter molecule 1 (Iba1), Intracerebroventricular (ICV), Indoleamine 2,3-dioxygenase-1 (IDO1), Interleukins (ILs), Intrathecal (IT), Lipid peroxidation (LPO), Malondialdehyde (MDA), Matrix metallopeptidase-13 (MMP-13), Mitogen-activated protein kinases (MAPKs), Nitric-oxide (NO), Nuclear factor-kappa B (NF-κB), Nuclear respiratory factor (NRF), Oral gavage (PO), Oxidative stress index (OSI), Superoxide dismutase (SOD), Spinal nerve ligation (SNL), Streptozotocin (STZ), Stimulator of interferon genes (STING), Total antioxidant status (TAS), Total oxidant status (TOS), Tumor necrosis factor alpha (TNF-α), Thiobarbituric acid reactive substances (TBARS).

**Table 3:** Carotenoids and derived compounds in inflammatory pain.

**Treatment**	**Induction Method**	**Animal/Dose**	**Effects**	**References**
Astaxanthin	CFA	Female rats, 25, 50, 100 mg/kg, PO, Days 15-28	↓ TNF-α, MDA, LPO, Nitrite, CRP, CCP ↑ GSH, CAT, SOD	[189]
		Male C57BL/6 mice 5, 10 mg/Kg, IP, 2 weeks	↓ p38, iNOS ↑ Nrf2, HO-1	[190]
Lutein		Male rats, 10 mg/Kg, IP,	↓ COX-2	[191]
Lycopene		Male rats, 10, 20 mg/Kg, PO, 4 weeks	↓ TNF-α, IL-1β, IL-2, IL-6, MCP-1, MIP-1α, MDA, NF-κB, P-P38, P-ERK, P-JNK/SAPK ↑ CAT, GSH-PX, Total SOD, Nrf2	[192]
Crocin		Rats, 40 mg/kg	↓ TNF-α, IL-1β, CRP, AST, ALT, ALP	[193]
		Male SPF rats, 160 mg/kg, PO, Days 15-28	↓ TNF-α, TGF-β1	[194]
Transcrocetin meglumine salt		Male C57BL/6 mice 10 mg/kg, IP, 3 days	↓ TNF-α, IL-1β, IL-6, Iba-1, GFAP	[195]
Safranal		Rats 200 mg/kg	↓ TNF-α, Urea, Creatinine, CRP, AST, ALT, Cox-2, MDA, NF-κB, ↑ GSH-PX,	[196]
Astaxanthin	Formalin	Male mice, 5, 10, 15 mg/kg, IP	↓ L-arginine, NO, Phosphodiesterase	[198]
Bixin		Male Swiss albino mice, 15, 30 mg/kg, PO,	MPO	[200]
Crocin		Male rats, 25, 50, 100, 200 mg/kg, IP, 30 minutes before induction of pain	Crocin increased morphine-induced antinociception	[201]
*Meso*-zeaxanthin	Formalin, Carrageenan, LPS-induced inflammation in macrophages	Male Balb/c mice, 50, 250 mg/kg, PO, 5 days before induction of pain	↓ TNFα, IL-1β, IL-6, CRP, iNOS, COX-2	[199]
Extract of the leaves of *W. heynei* (β-carotene Lycopene)	Formalin Carrageenan	Male and female rats, 150, 300 mg/kg, PO 1 hour before induction of pain	↓ TNFα, IL-1β, IL-6, PGE2, p-IKBα, p-KKβ, p-IKKα, p-p65	[203]
Astaxanthin	Carrageenan	Male ICR mice, 50, 100, 150 mg/kg	↓ MDA, ROS, MPO	[204]
Lycopene		Male rats, 1, 10, 25, 50 mg/kg, IP, 15 minutes	Anti-inflammatory effect	[205]
Crocin, Safranal		Male rats, 25, 50, 100 mg/kg, 0.5, 1 and 2 mg/kg, IP, 30 minutes before induction of pain	↓ Number of infiltrated neutrophils	[206]
Crocin		Male Kunming mice 12, 25, 50 mg/kg, PO, 1 hour before induction of pain RAW 264.7 murine macrophages 0.01, 0.1, 1, 10, 30, 100 μM	↓ PGE2, NF-κB, COX-1, COX-2	[207]
Lycopene	Capsaicin	Male Swiss albino mice 2 mg/kg, PO, 32 days	↓ Micronuclei formation, MDA, DNA fragmentation ↑ Ferric reducing ability of plasma.	[210]
Crocin		Male rats, 10 and 40 µg, ICV	Anti-nociceptive	[209]
FLEXPRO MD (Astaxanthin, Krill oil, and Hyaluronic acid)	LPS	Male C57BL/6 mice, 33, 67 mg/kg, PO, 2 weeks before injecting LPS RAW264.7 cells 10-100 μg/mL	↓ TNFα, IL-1β, IL-6, p-p65, p-IκB-α, iNOS, COX-2, MMP1, MMP2 ↑ IL-10	[218]

**Abbreviations**: Alanine aminotransferase (ALT), Alkaline phosphatase (ALP), Aspartate aminotransferase (AST), Catalase (CAT), Complete Freund’s adjuvant (CFA), Cyclic citrullinated peptide (CCP), C-reactive protein (CRP), Cyclooxygenase (COX), c-Jun N-terminal kinases (JNKs), Extracellular signal-regulated kinase (ERK), Glial fibrillary acidic protein (GFAP), Glutathione (GSH), Heme-oxygenase 1 (HO-1), Ionized calcium binding adaptor molecule 1 (Iba1), Intracerebroventricular injection (ICV), Interleukins (ILs), Inducible nitric oxide synthase (iNOS), Lipid peroxidation (LPO), Lipopolysaccharide (LPS), Matrix metallopeptidase (MMPMonocyte chemotactic protein 1(MCP-1), Macrophage inflammatory protein1α (MIP-1α), Myeloperoxidase activity (MPO), Nitric-oxide (NO), Nuclear factor-kappa B (NF-κB Prostaglandin E2 (PGE2 Reactive oxygen species (ROS), Specific pathogen-free (SPF), Superoxide dismutase (SOD), Stress-activated protein kinases (SAPK),, Transforming growth factor beta (TGF-β), Tumor necrosis factor alpha (TNF-α).

## LIST OF ABBREVIATIONS

ACC: Anterior Cingulate Cortex
ACh: Acetylcholine
AMD: Age-related Macular Degeneration
AP1: Activated Protein1
BDNF: Brain-derived Neurotrophic Factor
BSCB: Blood-spinal Cord Barrier
CCDs: Carotenoid Cleavage Dioxygenases
CCI: Chronic Constriction Injury
CIPN: Chemotherapy-induced Peripheral Neuropathy
CNS: Central Nervous System
CPSP: Central Post-stroke Pain
dlPFC: Dorsolateral Prefrontal Cortex
DRG: Dorsal Root Ganglion
ERKs: Extracellular Signal-regulated Kinases
GPx: Glutathione Peroxidase
hADSCs: Human Adipose-Derived Stem Cells
HIF-1α: Hypoxia-inducible Factor 1α
HIV: Human Immunodeficiency Virus
HO-1: Heme Oxygenase-1
HSV: Herpes Simplex Virus
ICV: Intracerebroventricular
LPS: Lipopolysaccharide
MCP-1: Monocyte Chemoattractant Protein 1
MDA: Malondialdehyde
MIF: Migration Inhibitory Factor
mPFC: Medial Prefrontal Cortex
MS: Multiple Sclerosis
NGF: Nerve Growth Factor
NO: Nitric Oxide
OSI: Oxidative Stress Index
ROS: Reactive Oxygen Species
SCI: Spinal Cord Injury
SOD: Superoxide Dismutase
TAS: Total Antioxidant Status
TBI: Traumatic Brain Injury
TLRs: Toll-like Receptors
TRP: Transient Receptor Potential
TRPA1: Transient Receptor Potential Ankyrin 1
VZV: Varicella-zoster Virus
